# Sulforaphane and Brain Health: From Pathways of Action to Effects on Specific Disorders

**DOI:** 10.3390/nu17081353

**Published:** 2025-04-15

**Authors:** Jed W. Fahey, Hua Liu, Holly Batt, Anita A. Panjwani, Petra Tsuji

**Affiliations:** 1 Department of Medicine, The Johns Hopkins University School of Medicine, Baltimore, MD 21205, USA; 2 Department of Psychiatry & Behavioral Sciences, The Johns Hopkins University School of Medicine, Baltimore, MD 21205, USA; 3 Department of Pharmacology and Molecular Sciences, The Johns Hopkins University School of Medicine, Baltimore, MD 21205, USA; 4 iMIND Institute, The Johns Hopkins University School of Medicine, Baltimore, MD 21205, USA; 5 Institute of Medicine, University of Maine, Orono, ME 04469, USA; 6 Stanley Division of Developmental Neurovirology, Department of Pediatrics, The Johns Hopkins University School of Medicine, Baltimore, MD 21205, USA; hliu8@jhmi.edu; 7 Anti-AGEs Foundation, Depew, NY 14043, USA; batt.hollyann@gmail.com; 8 Department of Nutrition Science, Purdue University, West Lafayette, IN 47907, USA; apanjwan@purdue.edu; 9 Center on Aging and the Life Course, Purdue University, West Lafayette, IN 47907, USA; 10 Department of Biological Sciences, Towson University, Towson, MD 21252, USA; ptsuji@towson.edu

**Keywords:** glucoraphanin, nutrition, broccoli, neurologic, psychiatric, autism, schizophrenia, detoxication, cognition

## Abstract

The brain accounts for about 2% of the body’s weight, but it consumes about 20% of the body’s energy at rest, primarily derived from ATP produced in mitochondria. The brain thus has a high mitochondrial density in its neurons because of its extensive energy demands for maintaining ion gradients, neurotransmission, and synaptic activity. The brain is also extremely susceptible to damage and dysregulation caused by inflammation (neuroinflammation) and oxidative stress. Many systemic challenges to the brain can be mitigated by the phytochemical sulforaphane (SF), which is particularly important in supporting mitochondrial function. SF or its biogenic precursor glucoraphanin, from broccoli seeds or sprouts, can confer neuroprotective and cognitive benefits via diverse physiological and biochemical mechanisms. SF is able to cross the blood–brain barrier as well as to protect it, and it mitigates the consequences of destructive neuroinflammation. It also protects against the neurotoxic effects of environmental pollutants, combats the tissue and cell damage wrought by advanced glycation end products (detoxication), and supports healthy glucose metabolism. These effects are applicable to individuals of all ages, from the developing brains in periconception and infancy, to cognitively, developmentally, and traumatically challenged brains, to those in later life as well as those who are suffering with multiple chronic conditions including Parkinson’s and Alzheimer’s diseases.

## 1. Introduction

**Figure 1:**
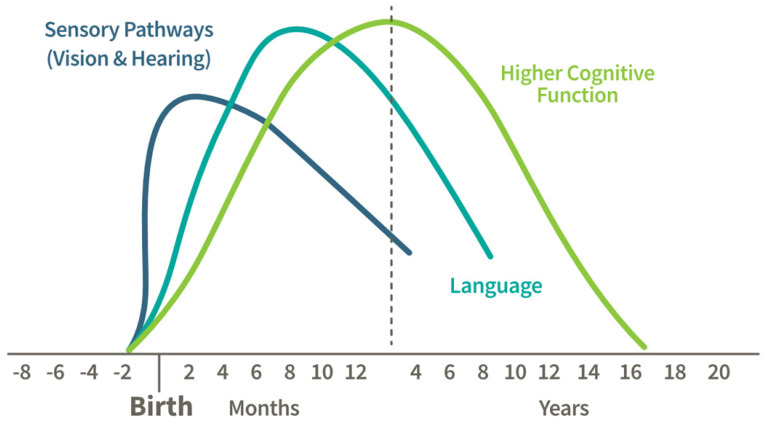
Human brain development. Sequential development of targeted neural pathways (synaptogenesis): First sensory (vision and hearing) synapse formation in the visual and auditory cortex, then language in the angular gyrus of the parietal lobe and speech production Broca’s area in the frontal lobe, then higher cognitive function in the prefrontal cortex. (Redrawn from [1]).

Brain development begins during the early stages of fetal development, with the formation of the neural tube in the first few weeks after conception, giving rise to the brain and spinal cord. By the second trimester, neurons begin to proliferate rapidly, forming the building blocks of the brain’s structure. During the third trimester and early postnatal period, synaptogenesis (the formation of connections between neurons) accelerates, laying the groundwork for sensory, motor, and cognitive abilities. Early childhood is marked by a surge in synaptic connections, known as synaptic overproduction, which supports learning and adaptation to the environment. This is followed by synaptic pruning, whereby unused connections are eliminated to improve neural efficiency. Adolescence is a critical period for the development of the prefrontal cortex, responsible for decision-making, impulse control, and complex reasoning. Myelination, the process of insulating neural pathways to enhance signal transmission, continues into early adulthood, enabling faster and more efficient communication between brain regions. Together, these processes shape the brain’s structure and function, supporting lifelong learning and adaptability [1] (Figure 1).

The brain is especially plastic and resilient to damage; therefore, cognitive decline often takes longer to develop, and symptoms typically manifest later in life compared to other chronic conditions, such as cardiovascular disease. Furthermore, the plasticity of the brain may allow it to effectively compensate for some deficiencies [2]. In fact, cognitive declines normally begin at the physiological level in humans starting in their 30s, but the symptoms of cognitive decline do not typically become measurable until the 40s and 50s, with declines in verbal fluency and other outcome measures. Symptoms of cognitive decline that negatively impact quality of life do not typically manifest until even later in life [3].

Therefore, it should not only be during adulthood and advancing years that people start becoming concerned about protecting and preserving brain function, but throughout life—from the periconceptional window to old age. We have the opportunity to intervene with diet and lifestyle choices that protect the developing brain, that foster its fullest and most thorough development, as well as help to protect it from neurological damage and degeneration as time marches forward. Because the same mechanisms that contribute to age-related cognitive decline (normal aging) overlap with those that contribute to neurodegenerative diseases, interventions that confer neuroprotective benefits for healthy individuals are often also relevant to disease states.

Nurturing, protecting, and supporting optimal brain health requires the consideration of a large suite of factors that include cognition, memory, and (neuro)protection against injury, inflammation, and oxidative stress. This, of course, also speaks to the entirety of the neurodevelopmental and neurodegenerative conditions that plague society. Micronutrients such as the familiar vitamins and minerals, as well as the critically important phytochemicals (also known as phytonutrients or bioactives)—present both an alternative and in some cases a complement to pharmaceuticals (discussed later in this review), for which development is extraordinarily expensive and slow. Brain-related pathways and mechanisms that are frequently targeted by phytochemicals, vitamins, and minerals include (1) central nervous system (CNS) activity, (2) cognition, (3) mood, (4) neuroprotection, (5) sleep, (6) stress, and (7) circadian rhythm. Support of ‘mental focus’, ‘concentration’, and ‘mental energy’ are among the many benefits sought by Western consumers of dietary supplements and healthy lifestyles. While most phytochemicals can fit into a daily routine either as foods or supplements, there is growing awareness that meditation, anxiety, sleep health, mood, stress, aging, and the prevention of dementia and Alzheimer’s disease are no longer just the niche for biohackers. Neuro- or “neurological support” is an all-embracing buzz word; however, neurologic target pathways are so vast that we must come to grips with the landscape prior to trying to outline solutions in which it appears that the phytochemical sulforaphane (SF) may have a role.

Early research on the biological activity SF was inspired, among other things, by observations from the epidemiologic literature on cancer prevention. In the 1970s, cruciferous vegetable consumption was linked to reduced incidence of a variety of cancers [4,5]. Over the years these associations have expanded to include a variety of conditions, including those impacting brain health. In the last half dozen years there have been epidemiologic studies showing, for example: a positive effect of vegetable consumption on psychological well-being [6]; significantly lower odds of experiencing high perceived stress associated with higher intakes of cruciferous vegetables [7]; an association between cruciferous vegetable consumption and sustained cognition in later life [8]; a link between higher intake of cruciferous vegetables and slower cognitive decline in middle-aged and older adults [9]; and beneficial effects of dark green and cruciferous vegetable consumption on cognitive function in older US adults, particularly regarding immediate and delayed learning abilities [10]. While various mechanisms have been invoked or speculated upon, these effects likely result from a constellation of protective effects. Notably, the presence of isothiocyanates (ITC) in cruciferous vegetables, in particular SF in broccoli, weaves a strong mechanistic thread through all of these epidemiologic findings.

**Figure 2:**
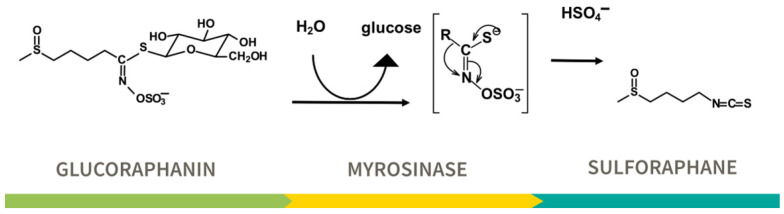
Glucoraphanin, myrosinase, and sulforaphane. Glucoraphanin is the biogenic, stable, and relatively non-biologically reactive glucosinolate precursor of sulforaphane. Glucoraphanin is converted to sulforaphane by myrosinase (E.C. 3.2.147), an enzyme not produced by humans, but present in their intestinal microbiome, as well as in each of the many thousand plant species that contain glucosinolates.

Among the bioactives, SF from broccoli is now one of the phytochemicals most rigorously supported by scientific studies—laboratory, animal, and human clinical trials. SF and other ITC from cruciferous vegetables exist in uninjured plants as glucosinolates. For SF, the cognate glucosinolate is called glucoraphanin (GR). GR is by and large not biologically active in its native form [11], but is converted to its biologically active SF by the enzyme myrosinase [12,13,14] (Figure 2). Myrosinase is also present in cruciferous plant cells and is normally segregated from glucosinolates until the cells are ruptured by chewing, freeze–thaw, cutting (food preparation), bruising, plant pathogens, or other damage.

Importantly this enzyme is also produced by a subset of the bacteria that populate the guts of all people studied to-date—our gastrointestinal microbiome [15,16,17,18]. Thus, when GR is ingested, myrosinase hydrolyzes it to SF, which is then rapidly conjugated with glutathione followed by stepwise hydrolysis of those conjugates, leading ultimately to N-acetylcysteine derivatives (mercapturic acids) that are excreted from the body. For purposes of clarity, we shall speak of SF (sulforaphane) in the discussions that follow. However, in most of the clinical studies the actual dosing agent was with plant (broccoli, broccoli sprouts, or broccoli seeds) extracts rich in GR, with or without added active myrosinase, and not SF since there are few if any stabilized SF preparations that are suitable for human ingestion. It should further be noted that for most of the laboratory (in vitro or ex vivo) studies and some of the animal model studies, it was actually SF that was used in dosing. The subtleties, issues, and concerns about bioavailability and dose selection are dealt with in great detail elsewhere and are beyond the scope of this review [13,19,20].

**Figure 3:**
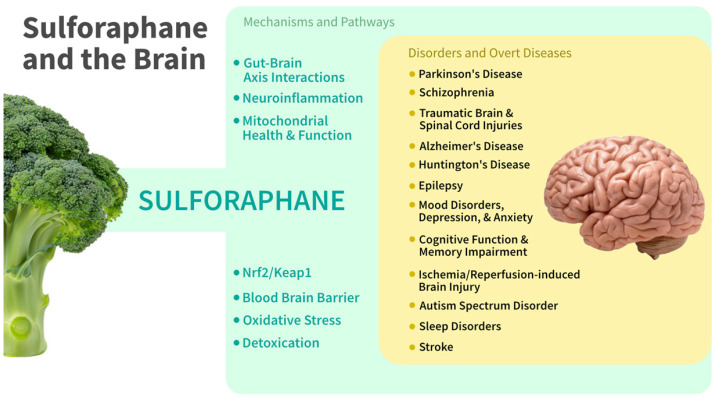
Sulforaphane and the brain. Sulforaphane has been shown experimentally and in many preclinical and clinical studies to act on a variety of pathways of cognition and brain health (*left side*), resulting in gradations of symptom amelioration, relief, or prevention of a variety of seemingly unrelated disorders and conditions (*right side*). In cases where cause and effect can be ascribed, pathways on the left side of this diagram have been linked to the effects of sulforaphane on those conditions by molecular, enzymatic, biomarker, or other biochemical evidence.

The role of SF in neurodevelopmental and neurodegenerative conditions has been reviewed [21,22,23,24,25]; however, the interconnectedness of mechanistic pathways and the number of specific points at which interventions may be effective, is extensive. These mechanistic points of access to the entire panoply of brain or cognition pathologies require further critical examination (Figure 3). A discussion of brain health and cognition also requires acknowledgement of the relationship between overall health status of the body and brain health. Most, if not all, factors that influence overall human health, directly impact the brain. A well-known example is high blood glucose, a risk factor for diabetes and cardiovascular disease, which is also a risk factor for cognitive impairment and dementia. Similarly, physical and psychosocial stressors that become chronic stressors also negatively impact the brain.

In Section 2, we discuss several neurologically relevant mechanistic pathways in which SF has the potential to confer neuroprotective and anti-neurotoxic effects in both the human brain and the CNS, across life stages. SF upregulates antioxidant and detoxification mechanisms, increasing brain glutathione (GSH), as well as the levels of a wide array of antioxidant and detoxification proteins (enzymes). Among neuroprotective mechanisms we review are the upregulation of autophagy and proteasomal regulation leading to reductions in protein miscoding, misfolding, and the removal of damaging protein aggregates as well as the modulation of and protection against neuroinflammation. Also reviewed is the active protection by SF of neuronal cell mitochondrial function and metabolism and protection from mitochondrial damage. SF can boost the activity of several critical detoxification mechanisms, protecting against amyloid beta (Aβ) fragment cytotoxicity, perturbed calcium homeostasis, excitotoxicity, and neurotoxin damage. SF can be highly effective in the preservation of the integrity and function of the blood–brain barrier (BBB), to which it is permeable. And SF can protect against neuronal cell death by inhibiting apoptosis, by upregulating brain-derived neurotrophic factor (BDNF) it can enhance neuronal function and plasticity, and support neurogenesis.

In Section 3, we then profile several of the disorders, conditions, or diseases for which there is most evidence or for which the existing evidence is most compelling for the ameliorative or preventive role of SF.

## 2. Pathways Through Which SF Has Been Shown to Impact Brain Health and Cognition

Note that whereas ‘normal’ aging is considered as a treatable quasi-disease state by some and is actively looked upon as something treatable with non-pharmacologic interventions [26], it is embraced in many of these categories. The authors of the present review do not approach aging as a disease.

### 2.1. The Blood–Brain Barrier (BBB)

**Figure 4:**
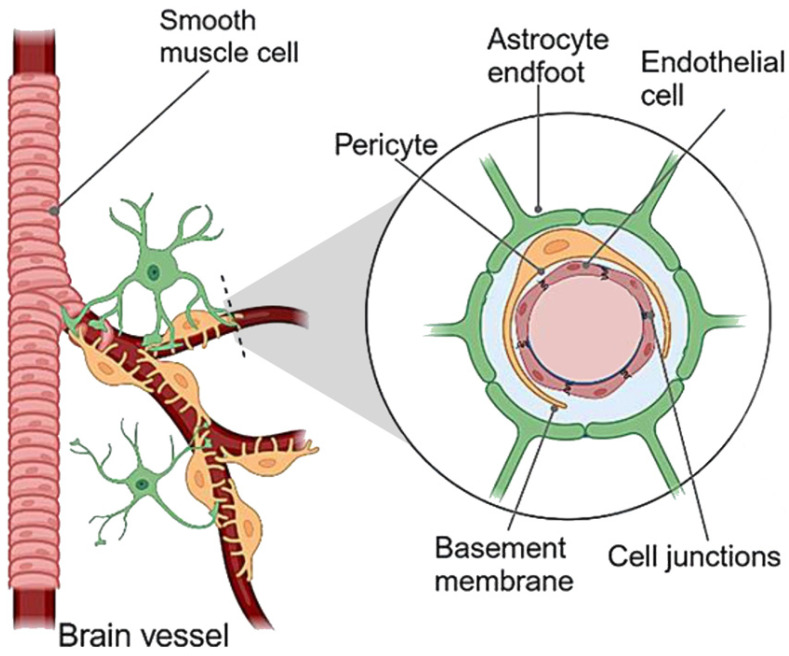
Structure of the blood–brain barrier (BBB). The BBB is composed of several cell types; the smooth muscle cells are present in vessels with higher diameters, astrocytes bind to the vessel through their feet, and pericytes enclose the vessel and are surrounded by a basement membrane. The endothelial cells are tightly bound together through several cell junction proteins, which include claudin and occludin that in turn connect to the actin cytoskeleton through zonulin, VE-Cadherin, and other permeability-modulating proteins (adapted from Cazalla 2024; ref. [25]).

The BBB is a critically important physico-chemical mechanism protecting the brain against cellular and environment-derived toxins and other damaging compounds. It is created by a consortium of endothelial, nerve, and smooth muscle cells forming the walls of the capillaries supplying the brain, and it thus covers a huge surface area for exchange “across”, “in”, or “into” the brain—estimated to be between 12 and 18 m^2^ in adults [27]. Across this interface, there is: (a) a physical barrier formed by tight junctions between cells (visualized in Figure 4), (b) a barrier to transport, as a result of ion channels and other means of modulating solute flux, and (c) a metabolic barrier created by a suite of enzymes, which metabolize molecules that they contact. The BBB is modulated and regulated both physiologically and pathologically in a multitude of ways since it is composed of epithelial cells, neuronal cells, and smooth muscle cells. Its capacities include controlling the flux of small molecules, metabolites, and even other cells (e.g., neutrophils and mononuclear cells that are attracted to sites of BBB inflammation); providing protection from pathogens and the inflammation and disease they may bring; regulating cerebral blood flow; it is critical to clearing metabolic waste and other products of brain activity; providing a stable microenvironment and maintains homeostasis requisite for complex neural function; releasing diffusible signals, such as nitric oxide, prostanoids, and ions that in turn regulate blood flow [27].

Any discussion of a compound’s effect on brain health should first examine whether- and how that compound interacts with the BBB. Disruption of this physically separating interface allows for the unregulated uptake of toxins and other damaging compounds to enter the CNS and is a hallmark of neuronal damage, age-related cognitive deficits, and neurological disorders. In fact, among other recent developments in delivering SF to the brain is its capacity to cross the BBB and accumulate in CNS tissues [28,29], and SF is also the object of efforts to develop multimeric drug combinations for the treatment of glioma that are ‘led through’ the BBB as self-assembling nanomicelles by SF [30].

The prevention of BBB dysfunction is one of the key mechanisms of action through which SF exerts its neuroprotective effects [31]. The primary way SF protects BBB integrity appears to be through its activation of the Kelch-like ECH-associated protein 1 (KEAP1)/nuclear factor erythroid 2-related factor 2 (NRF2) pathway, since BBB integrity is rapidly compromised in the absence of Nrf2 transcriptional activity [32]. SF also preserves tight junction alterations via anti-inflammatory and anti-apoptotic activities [22]. For example, in a mouse stroke model, pre-treatment with SF (5 mg/kg i.p.) significantly improved neurobehavioral deficits, reduced lesion progression and attenuated BBB disruption [33]. These protective effects were associated with enhanced antioxidant defenses including upregulation of the expression of Nrf2 and heme oxygenase-1 (HO-1) in the brain. Similarly, in a vascular cognitive impairment model that mimics the effects of chronic cerebral ischemia, treatment with SF (10 mg/kg twice a week) improved several outcomes compared to the control group. It preserved the integrity of the BBB via Nrf2 as evidenced by increased expression of HO-1. This model simulates the human conditions of restricted blood flow and subsequent cognitive impairment due to carotid artery plaque build-up. In the control group, chronic deficits in sensorimotor coordination and balance function as well as impairments in learning, memory, and spatial cognitive functions were observed. In contrast, the SF treatment group showed attenuated deficits, along with reductions in neuronal death, myelin loss, abnormal axons, and white matter injury [34].

### 2.2. The Keap1/Nrf2 Pathway, Detoxication, and Oxidative Stress

**Figure 5:**
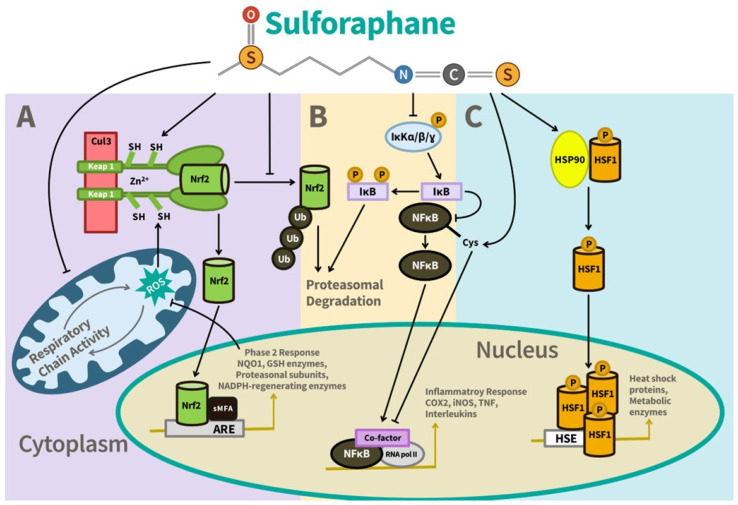
Major biochemical pathways that are affected by sulforaphane (SF). (**A**) SF upregulates the Nrf2 pathway via its binding to the chaperone protein Keap1, with attendant interactions with mitochondria; (**B**) SF inhibits the NF-кB pathway through its interaction with IкK or cysteine residues in NF-кB; (**C**) SF upregulates the heat shock proteins (HSP). (redrawn from Liu 2016; ref. [14]).

It is well accepted that oxidative stress is associated with damage to the CNS and that high rates of energy consumption, ~20% of whole-body oxygen consumption despite its small size (ca. 2% of body mass), make the brain especially susceptible to oxidative damage [35]. The high lipid content of the brain, especially its polyunsaturated fatty acid content, increases its vulnerability to lipid peroxidation [36]. Exposure to environmental and cellular derived toxicants damages the brain directly and further exacerbates oxidative and inflammatory injury. Therefore, maintenance of antioxidant status and redox balance within the brain is a key component of neuroprotection. Fortunately, several protective mechanisms exist to protect the brain against these damaging insults, including antioxidant and detoxification pathways, as well as physical barriers like the BBB. One of the most well-understood and extensively studied pathways is the Keap1/Nrf2 pathway (Figure 5). Nrf2 is a highly inducible transcription factor activated by SF that regulates the basal and inducible expression of a large battery of between 250 and 500 genes, accounting for 3–5% of total cellular protein transcription [32,37,38] (Table 1).

**Figure 6:**
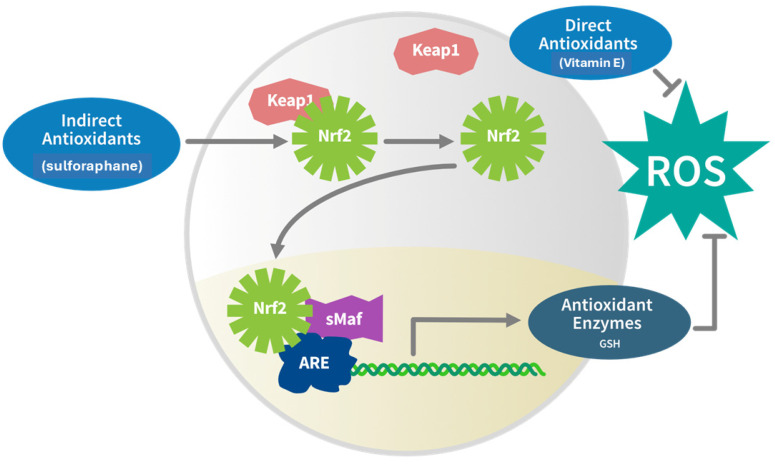
Indirect antioxidant strategy. Direct antioxidants like vitamins E (*right side*) and C act directly to neutralize reactive oxygen species (ROS) and reactive nitrogen species. Indirect antioxidants like SF (*left side*) are potent inducers of the cells’ antioxidant enzymes as well as the body’s most abundant endogenous antioxidant, glutathione (GSH), which together act to neutralize ROS and other oxidants. By virtue of the fact that these are enzymes (and GSH is continuously enzymatically re-reduced and made re-available), this effect of SF is long-lived (hours to days) as opposed to direct antioxidants, which are used up as soon as they act.

These genes encode for cytoprotective factors including those that defend against electrophilic stressors, toxins, chemical carcinogens, and direct oxidative stressors [39]. As a result, Nrf2 inducers like SF that have no direct redox activity are often referred to as “indirect antioxidants” [40,41,42] (Figure 6). This makes Nrf2 an attractive target for the development of both drugs and natural products [43,44,45,46]. It has been established that Nrf2 is normally sequestered in the cellular cytoplasm by the chaperone protein Keap1 and several other regulatory elements [47]. Upon reaction with SF or other inducers, Keap1 alters its conformation, and releases Nrf2, which then travels to the cell’s nucleus where it binds to the Antioxidant Response Element (ARE) and triggers the coordinated transcription of genes that regulate detoxification and cellular redox balance. Among the up to 500 genes that SF can upregulate through the Keap1/Nrf2/ARE signaling pathway are the antioxidant and detoxification enzymes NAD(P)H:quinone oxidoreductase-1 (NQO1) and heme oxygenase-1 (HO-1 or HMOX), profiled in the following section, as well as catalase (Cat), superoxide dismutase(s) (SOD), peroxiredoxins (Prx), heat shock proteins (HSP), glutathione *S*-transferases (GST), thioredoxin reductase (Trx), glutathione synthetase (GS), glutathione peroxidases (GPx) and glutathione reductase in the brain [38,48]. These proteins (enzymes) and their interconnected detoxication pathways are critical in protecting the brain against environmental toxins against cell-generated toxic molecules, and against advanced glycation end-products (AGEs) associated with poor diet and lifestyle, which are closely linked to the development of diabetes and related health issues [49].

#### 2.2.1. Glutathione (GSH)

GSH is one of the most important endogenous antioxidants within the brain, and it is the most ubiquitous and abundant intrinsic non-enzymatic antioxidant in the human body [50]. Whereas other indirect antioxidants in the brain such as ascorbic acid are present in the μM range, GSH concentrations in healthy human brain are much higher, in the range of ~1–2 mM [51]. GSH plays a key role in the regulation and maintenance of intracellular redox status in the brain as well as participating in multiple detoxification functions [52,53]. For example, it irreversibly binds toxic electrophiles in a reaction catalyzed by GST, depleting the cell of two GSH molecules in the process, but rendering the electrophiles inert so that they may be excreted from cells and tissues without causing further damage [54]. In addition to these systemic protective functions, GSH serves several beneficial roles specific to the brain. It contributes to maintenance of the integrity of the BBB by protecting against oxidative injury [51,55]. Acting as a cofactor in the glyoxalase enzyme system in neurons and glial cells, GSH is responsible for the detoxification of methylglyoxal, preventing the production of damaging AGEs [51,53,56]. GSH is also responsible for the detoxification of acrolein, a toxic byproduct of polyamines in the brain [57]. Moreover, GSH plays an important role in protecting neuronal cells against nitrosative stress, preventing the production of the neurotoxic and pro-inflammatory compound peroxynitrite in a reaction catalyzed by GPx [55,58]. Deficits in brain GSH concentrations are associated with increases in oxidative damage [59,60], impaired ability to quench and export toxins [57,61], neuroinflammation [62], and increased NO toxicity and neuronal cell death [52].

The ability of SF to upregulate GSH in the brain is critical for antioxidant protection in youth but may become even more important with age. This is because brain GSH concentrations are depleted not only in a variety of neurodegenerative disorders, but also in normal aging [63]. In models of healthy aging, GSH concentrations have been consistently demonstrated to decline by as much as 50% in brains of older rodents compared to younger ones [36,57,59,60,64,65,66]. A single cross-sectional study examining GSH levels in healthy humans revealed decreased GSH concentrations directly in the occipital cortex of older (76.6 ± 6.1 years) participants, as compared to younger (20.4 ± 1.4 years) subjects [67]. Depletion of brain GSH has been demonstrated to disrupt short-term spatial recognition, learning and memory and cognitive flexibility in rodents [68,69], and to exacerbate stroke infarct size in mice [57] and in humans [61].

**Figure 7:**
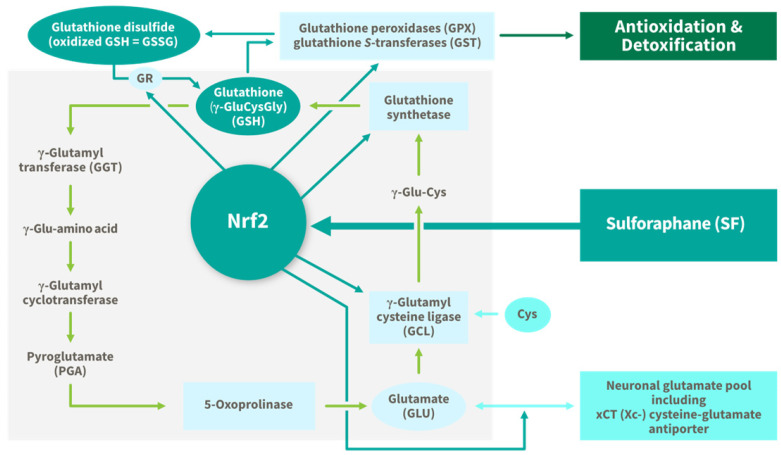
Interactions of sulforaphane and Nrf2 with the gamma-glutamyl (glutathione; GSH) cycle. Additional abbreviations used for clarity in this figure: Cys, cysteine; GR, glutathione reductase.

SF has been consistently demonstrated to increase the synthesis of GSH in diverse tissues including within the brain and CNS [70,71,72] via the Keap1/Nrf2 pathway by upregulating the enzymes involved in the synthesis, recycling, and sparing of GSH: γ-glutamylcysteine ligase (γ-GCL), γ-glutamylcysteine ligase catalytic subunit (GCLC), γ-glutamylcysteine ligase modifier subunit (GCLM), GST, GPx, GS, and glutathione reductase. SF induces the expression of both rate limiting enzymes involved in GSH synthesis, GCLC and GCLM, increasing the production of GSH in brain cells directly (Figure 7) [22]. In addition to inducing the synthesis of GSH, a large body of evidence supports the role of SF in maintaining the redox balance of GSH by promoting the synthesis of enzymes that recycle oxidized GSH (GSSG) back to its active, reduced state (GSH) [70] (Figure 7). In fact, oral supplementation with 100 μmol SF derived from broccoli sprout extract has been reported to increase GSH levels in situ in the brains of healthy human subjects (*n* = 9), assessed by magnetic resonance spectroscopy [70]. Moreover, increased plasma GSH levels correlated with concentrations in the brain, suggesting that peripheral GSH status may be a proxy for brain GSH status. In the SF treatment arm of an intervention with 57 children with autism, there were lower ratios of both free GSH to GSSG, and total GSH to free GSSG in the blood than in the placebo group, at both 15 and 30 weeks of intervention [71]. Therefore, the fact that oral supplementation with SF upregulated GSH levels directly in the brain of healthy humans suggests increased synthesis of GSH. These data indicate a powerful neuroprotective potential of SF for maintaining or restoring GSH and for protecting neurons, specifically, and more broadly, the entire CNS.

As neurons do not have a mechanism to take up GSH, they must rely on de novo synthesis and are therefore especially vulnerable to age-related disruptions in GSH synthesis [51]. Reductions in de novo synthesis of GSH is considered one of the primary factors responsible for impaired brain GSH concentrations with advanced age. Decreased levels of both the GCLC and GCLM regulatory subunits of γ-GCL have been reported in the brains of aged mice, in the absence of changes in GS activity [57]. Furthermore, these reductions in de novo GSH synthesis in the brain preceded age-related cognitive decline associated with GSH deficiency [57]. SF has been consistently demonstrated to induce the expression of both GCLC and GCLM in neural cells offering further evidence for its utility to maintain or improve GSH status [22].

Another contributing factor leading to increased depletion of GSH with age is related to increased oxidative and nitrosative stress, toxin levels, and neuroinflammation. In addition to its antioxidant function, GSH plays an essential role in detoxification reactions, irreversibly binding toxic electrophiles and environmental toxicants, a process which depletes GSH levels. Therefore, cumulative exposure to toxins that occurs with aging results in greater depletion of GSH within the brain. And finally, increased expression of multidrug resistance transporter proteins in response to oxidative stress increases the export of GSH out of cells, including from endothelial cells of the BBB, further depleting neuronal GSH [55].

#### 2.2.2. NAD(P)H:Quinone Oxidoreductase-1 (NQO1; Previously Called Quinone Reductase or QR)

NQO1 has critical cellular antioxidant activity, directly scavenging superoxide radicals, as well as exerting indirect antioxidant effects by regenerating the antioxidants GSH, CoenzymeQ_10_, and alpha-tocopherol, all of which play critical roles in neuroprotection, and together contribute to maintenance of the redox balance of the brain and CNS [73]. In addition to its enzymatic activities, NQO1 is involved in the recognition, repair, and removal of damaged proteins. NQO1 prevents the proteasomal degradation of the tumor suppressors p53, p73α, p33, and other important cytoprotective proteins [74]. NQO1 plays a protective role against the genotoxicity of the procarcinogen benzene and its benzoquinone metabolites [75]. It has been proposed that the direct ability of NQO1 to catalyze the detoxification of quinone metabolites makes it a critical component of cellular defense against neurotoxicity [48]. SF increases NQO1 gene and protein expression through the Nrf2 signaling pathway in most tissues examined, including within the CNS [22]. SF administration to astrocytes increased NQO1 concentrations and protected against oxygen and glucose-induced astrocyte cell death in a primary cell culture model [76]. Similarly, SF protected hippocampal neurons against heme-, oxygen-, and glucose toxicity (death) via increased expression of GCLM, NQO1, and HO-1 [77]. Early work in Paul Talalay’s lab at Johns Hopkins identified NQO1 as a key and highly responsive Phase 2 enzyme, the measurement of which has been a reliable and highly predictive indicator of the cytoprotective potential of both natural and synthetic compounds [78]. The Talalay lab then developed a microtiter plate based quantitative assay for monitoring NQO1 activity. For over 30 years, it has been a workhorse in that laboratory and has been used worldwide to great advantage [79]. Even very recently, expression of NQO1 in peripheral blood mononuclear cells has been used as a proxy for the expression of the whole suite of Keap1/Nrf2 regulated protective enzymes in human clinical studies [80,81]. For example, Ushida et al. reported dose dependent increases in NQO1 and GST activity in serum following, single, acute doses of either 30 mg or 60 mg GR from broccoli sprout extract in 21 healthy men and women [76].

#### 2.2.3. Heme Oxygenase-1 (HO-1; HMOX; HSP32)

HO-1 is an enzyme with well-established neuroprotective effects [34] that were linked to the phase 2 antioxidant and detoxication phenomenon by the Talalay lab as a key and highly inducible phase 2 chemoprotective enzyme [78,82]. Since then, its activation has been well documented to be a crucial mechanism for stress response in the nervous system, where its constitutive levels are quite low, but it can be highly induced [37,83]. HO-1 protects cells through its antioxidant functionality, specifically targeting the superoxide radical and other reactive oxygen species [22]. In addition, HO-1 modulates inflammatory responses, providing important endogenous defenses against oxidative and inflammatory injury in the brain [33]. As just two of many examples, treatment of rats with SF (5 mg/kg i.p.) enhanced antioxidant defenses, upregulated the expression of Nrf2, HO-1, and Prx in the brain within 24 h compared to untreated controls [33]; and in an accelerated aging mouse model SF prevented age-related cognitive decline by maintaining mitochondrial function and biogenesis and robustly upregulating a suite of Nrf2-regulated genes, including those for HO-1, in the hippocampus [84].

### 2.3. Detoxication

Detoxification, or detoxication as it has been our convention to refer to the process, involves the removal or neutralization of toxicants (xenobiotics)—toxic molecules from the environment, food, or endogenously produced compounds. Xenobiotic metabolism comprises three phases: Phase 1—primarily accomplished through many enzymes of the cytochromes P450 family, which generally introduce polar groups or reactive groups to xenobiotics; in Phase 2, these modified xenobiotics as well as other non-modified compounds are conjugated to polar compounds in particular to GSH—reactions that are up-regulated by the Keap1/Nrf2 pathway (see Table 1 and Figure 5); and in Phase 3, metabolism can involve further chemical modification and removal from the cells by efflux transporters, followed of course by excretion from the body in sweat, stool, breath, or urine. One exception to this statement may be microplastics, which have just been estimated from a series of 91 cadaver samples to accumulate to levels of >5 g per brain, to be climbing rapidly over time, and to average more than 5 times higher than this in the brains of persons with dementia (*n* = 12) [85,86]. SF is one of the most potent facilitators of detoxication through Phase 2 metabolism, promoting the detoxication of a wide variety of environmental and food-based pollutants [87] through its action in inducing or boosting the Keap1/Nrf2 pathway (Table 2). We have focused upon it here in a separate section because it is such a critical process both for systemic protection and neurologic health.

Environmental pollution has been linked to many negative health outcomes including shortened lifespan and increased chronic disease, but notably also include a variety of neurodevelopmental problems, neurotoxicities, and increased risk of dementia. There are now hundreds of papers that deal with the detoxifying capacity of SF, and there are well over a thousand studies focused on Nrf2 from a detoxication perspective. Clearly, we cannot review them all herein, so we will focus on a diverse array of clinical studies that some of the authors have been involved with, which exemplifies the broad range of SF’s detoxication capacities.

In the 1990s and early 2000s, a number of groundbreaking preclinical and clinical studies were conducted on the detoxication of the potent liver carcinogen and toxin, aflatoxin [88,89]. Aflatoxin B1 is also a potent neurotoxin. It crosses the BBB and leads to a host of neurodevelopmental and neurologic disorders in addition to its effects on growth retardation and its potent carcinogenicity [90,91]. SF from broccoli sprouts was then utilized to demonstrate aflatoxin conjugation and enhanced excretion from the body [92]. This was a randomized placebo-controlled 2-week intervention with 200 free-living healthy subjects in rural China where the aflatoxin exposure was extraordinarily high, as was the prevalence of liver cancer. There was a highly significant inverse association (*p* < 0.002) between urinary levels of the aflatoxin-DNA adduct aflatoxin-N^7^-guanine and levels of dithiocarbamate, a biomarker of SF bioavailability. This suggested that SF was exerting a pharmacodynamic action that reflected induction of GSTs by SF, thereby shunting the reactive epoxide intermediate away from nucleophiles in DNA, where it would have mutagenic and carcinogenic effects, and toward GSH, which allowed it to be further metabolized and excreted in the urine. Furthermore, a metabolite of the combustion product *trans,anti*-phenanthrene tetraol was also highly inversely correlated with dithiocarbamate levels (*p* = 0.0001). This compound is a metabolite of the combustion product phenanthrene, reflecting exposure to the well-established environmental carcinogens polycyclic aromatic hydrocarbons (PAH). This secondary endpoint then pointed the team to further studies on the capacity of SF to detoxify air pollutants, since these levels were continuing to rise in the study area whilst levels of aflatoxins (and liver cancer) were falling. Subsequent clinical studies then turned to SF-facilitated detoxication of these air pollutants [90].

**Table 2:** Detoxication targets of SF.

Type of Compound	Neurotoxin	Reference
advanced glycation endproducts (AGE) precursor	methylglyoxal	[49]
air pollutant	diesel exhaust	[93]
air pollutant	cigarette smoke	[94]
air pollutants from combustion and tobacco smoke	benzene, acrolein	[95,96,97,98]
antimuscarinic alkaloid	scopalamine	[99]
dinoflagellate-derived shellfish toxin	okadic acid	[100]
DNA alkylating antibiotic	streptozocin	[101]
fungicide	pyraclostrobin	[102]
fungicide	trifloxystrobin	[102]
fungicide	famoxadone	[102]
fungicide	fenamidone	[102]
heavy metal	Cadmium	[103]
heavy metal	Arsenic	[104]
heavy metal	lead	[105]
polycyclic aromatic hydrocarbons (PAH; air pollutants)	phenanthrene	[92]
pesticide	rotenone	[102,106]
pesticide	tributyltin	[107]
pesticide	paraquat	[108]
poisonous gas	carbon monoxide	[109]
spoiled grain contaminant	aflatoxin	[88]
street drug	methamphetamine	[110,111]
street drug	phencyclidine	[112,113]
street drug contaminant	MPTP	[114]
toxic pathogen-associated molecular patterns (PAMP)	bacterial lipopolysaccharide	[115]

Air pollution is associated with reductions in cognitive performance at younger ages, and it predisposes people to dementia. For example, declines in cognitive performance in verbal and math tests have been reported in subjects under chronic exposure to air pollution, with longer exposure leading to greater deficits [116]. Several large-scale clinical trials have reported that supplementation with GR and/or SF increases urinary excretion of environmental air pollutants, providing evidence of a protective role of SF against damage. Participants were supplemented with 800 μmol GR and/or 150 μmol of SF for one week, which led to a significant increase in the urinary excretion of the environmental air pollutants benzene and acrolein [95]. These findings were confirmed in a subsequent study in which supplementation of 291 subjects with a combination of GR (614 μmol) + SF (40.5 μmol) for 12 weeks resulted in significant increases in the urinary excretion of benzene and acrolein [97]. More recently, Chen and colleagues randomized 170 adults to one of three doses of GR + SF: (1) 125 μmol GR + 8 μmol SF, (2) 300 μmol GR + 20 μmol SF, or (3) 600 μmol GR + 40 μmol SF for 10 days [96]. SF and SF metabolites increased in the urine in a dose dependent manner, although benzene excretion in the urine was significantly increased only in the highest dose group. These data confirmed that SF effectively increases urinary excretion of environmental pollutants in humans, reducing exposure and accumulation of these neurotoxic chemicals in a dose-dependent manner.

Tobacco smoke, in addition to its clear addictive effects, has a significant impact on neurodevelopment, neurotransmission, and cognitive function, as well as promoting the course of neurodegenerative and cerebrovascular diseases and even insomnia [117]. Similarly, diesel exhaust exposure can disrupt autophagy, and has been linked to higher levels of Parkinson’s Disease [118]. Functional MRI has been used to show that even brief exposure to diesel exhaust smoke can have profound effects upon functional brain connectivity [119]. The effects of SF to ameliorate these two major sources of indoor and environmental air pollution have been evaluated in clinical studies using a variety of biomarkers [93,94,98]. An additional hazard that smokers are at greater risk of than non-smokers is influenza infection. This is thought to be because their nasal mucosal interleukin 6 (IL-6) and interferon γ-induced protein 10 (IP-10) responses influenza virus are diminished. Thus, short-term ingestion of broccoli sprout homogenates by smokers significantly reduced virus-induced markers of inflammation, as well as reducing virus quantity following a challenge with a live attenuated influenza virus vaccine [120].

In addition to its protective effects against airborne pollutants, SF defends against methylglyoxal toxicity [56,121,122] in cultured neural cells. Methylglyoxal, a precursor of AGEs, poses a particular threat to neural tissue, and both of these toxic compounds have been found in the brains of people with Alzheimer’s disease. Intervention with SF can mitigate this danger [49]. SF may also prevent or slow the process of normal brain aging and memory problems as demonstrated in neuronal cell and animal models exposed to various toxins, including pesticides like rotenone, which are linked to an increased risk of Parkinson’s disease. It also appears to mitigate the effects of fungicides, which cause transcriptional changes similar to those observed in brain samples from individuals with autism, advanced age, Alzheimer’s disease, and Huntington’s disease [102]. These are listed in Table 2.

### 2.4. Mitochondrial Health and Function

The human brain has especially high energy demands and therefore relies heavily on mitochondrial energy production. As mentioned previously, the brain utilizes ~20% of whole-body oxygen consumption despite making up only 2% of body mass, making it especially vulnerable to oxidative damage and environmental toxins, but also to energy depletion [123,124]. Not surprisingly, age-related deficits of GSH in brain, including deficits in brain mitochondrial fractions, correspond with impairments in mitochondrial function and energy production [125]. Mitochondrial dysfunction and damage are implicated in the cognitive decline associated with normal aging as well as in the pathogenesis of most neurodegenerative disorders [126]. All evidence indicates that the Nrf2 pathway is intimately involved in several aspects of mitochondrial function and metabolism [126], playing a pivotal role in maintaining mitochondrial health and cellular metabolism, especially in the context of neurodegenerative diseases. Dysregulation of mitochondrial function and energy metabolism are hallmarks of disorders like Parkinson’s and Alzheimer’s, making Nrf2 activation a promising therapeutic target for phytochemical or nutritional support.

Nrf2 contributes to mitochondrial bioenergetics by enhancing mitochondrial membrane potential, substrate availability for respiration, and ATP production—crucial for energy-demanding neurons. It also regulates enzymes involved in glucose metabolism, the tricarboxylic acid (TCA) cycle, and the pyruvate dehydrogenase complex, which facilitates the entry of pyruvate into mitochondria. Additionally, Nrf2 modulates malic enzyme 1 to generate NADPH, which is vital for oxidative stress defense and neurotransmitter cycling in astrocytes. In neurodegenerative conditions where fatty acid metabolism is impaired, the effective modulation of this pathway ensures efficient energy production from mitochondrial fatty acid oxidation [126].

As a potent Nrf2 activator, SF enhances mitochondrial function through several mechanisms. SF has been shown to prevent tert-butyl hydroperoxide-induced mitochondrial permeability transition pore opening in rat brain and liver mitochondria, and to protect against mitochondrial dysfunction caused by lipid peroxidation byproducts like 4-hydroxynonenal [127]. In animal models, SF enhances mitochondrial function in the hippocampus, reduces neuronal damage in seizure models, and mitigates chemically or electrically induced mitochondrial dysfunction [128]. In a study with senescence-accelerated mice and normal aging controls, treatment with SF for about a year significantly improved long-term memory and increased hippocampal mRNA and protein levels of peroxisome proliferator-activated receptor gamma coactivator-1 alpha (PGC1α) and mitochondrial transcription factor A (TFAM), both of which are master regulators of mitochondrial biogenesis. Additionally, SF treatment elevated mRNA levels of nuclear respiratory factor-1 (NRF-1) and mitochondrial DNA-encoded respiratory complex enzymes [84].

Beyond these primary effects, SF produces a range of other mitochondrial benefits. It stimulates mitochondrial biogenesis, maintains respiratory complex activity, and enhances bioenergetic parameters, including oxygen consumption [129]. A recent clinical trial with SF treatment on autism spectrum disorder (ASD) by Zimmerman et al. demonstrated changes in mitochondrial function that correlate with ASD behavior scores [71]. In this clinical study, changes in the Aberrant Behavior Checklist (ABC) scores were significantly related to change in ATP-Linked Respiration and Proton-Leak Respiration in the 27 subjects who were able to have their mitochondrial function evaluated (Ref. [71]; discussed in more depth in the section of this review on ASD).

Mitochondrial protection through the Nrf2 pathway also supports resilience against a wide array of neurotoxins, which can disrupt oxidative phosphorylation and ATP production. Genes promoting mitochondrial biogenesis, maintaining complex I, II, and IV function, and preventing ATP depletion caused by toxins can all be upregulated by the Keap1/Nrf2 pathway. This broad neuroprotective scope of SF includes reduced damage across models of Huntington’s disease, epilepsy, and chemotherapy-induced neuropathy, as well as exposure to carbon monoxide and various environmental toxins as summarized in Table 2.

By modulating mitochondrial redox homeostasis, respiratory activity, and energy production, SF protects neurons from oxidative stress, excitotoxicity, and apoptosis, positioning it as a versatile agent against the effects of environmental and pharmaceutical neurotoxins. As a potent Nrf2 activator, SF addresses both systemic and brain-specific oxidative stress and inflammation as well as improving mitochondrial bioenergetics and metabolic resilience.

### 2.5. Neuroinflammation

It is well known that chronic inflammation plays a central role in aging and age-related diseases including diabetes, cardiovascular disease, autoimmune disorders, etc. Similarly, neuroinflammation is a ubiquitous risk factor for cognitive-related conditions including age-related cognitive decline, dementia, stroke, and Alzheimer’s disease. In fact, neuroinflammation is now considered a predisposing risk factor for learning and memory impairment [21], and elevated neuroinflammation and oxidative stress, driven by increased microglial activation, are linked to age-related cognitive decline [130]. It has been established that the Nrf2 pathway plays a crucial role in defending against inflammation, including neuroinflammation. Activation of the phase 2 enzyme, HO-1, and suppression of the nuclear factor-kappa B (NF-kB) signaling pathway in the CNS through Nrf2 are both highly effective mechanisms targeted by known Nrf2 inducers like SF to combat neuroinflammation. Several lines of research have documented the inflammatory modulating effects of SF, particularly its ability to block the NF-kB pro-inflammation pathway (Figure 5), which has been the subject of extensive study both in vitro and in humans [21,37,39,71,80,94,131,132,133,134,135,136,137,138,139]. These and other studies have demonstrated that SF can reduce the levels of pro-inflammatory cytokines IL-6, IL-1β, TNF-α, and the pro-inflammatory lipid mediator Prostaglandin E2 (PGE2), as well as the expression of the pro-inflammatory enzyme cyclooxygenase 2 (COX-2). Additionally, SF inactivates the pro-inflammatory cytokine Macrophage Migration Inhibitory Factor (MIF) and suppresses the production of nitric oxide. The regulation of target proteins and metabolic pathways in neuronal cells by SF led to decreased nuclear translocation of NF-kB, thereby lowering the production of key pro-inflammatory mediators, cytokines, and oxidative markers that drive neuronal apoptosis [31].

A lipopolysaccharide (LPS)-induced inflammation model has been frequently used to study inflammation. Of particular relevance to this review, this model was used to study cognition and memory impairment in mice. SF pretreatment (20 mg/kg, administered i.p. for 7 days) reversed LPS-induced cognitive and memory deficits, as demonstrated by improved performance in Morris water maze tests [140]. Specifically, SF mitigated LPS-induced impairments, such as reduced platform crossings, shorter time spent in the target quadrant, and increased escape latency and path length. These improvements were ascribed to SF’s ability to attenuate LPS-induced pro-inflammatory cytokine production, restore synaptic protein expression, and normalize the BDNF-tropomyosin receptor kinase B (TrkB)-mechanistic target of rapamycin (mTOR) signaling in the hippocampus. Notably, inhibition of TrkB-mTOR signaling by the TrkB inhibitor ANA-12 or the mTOR inhibitor rapamycin nullified the cognitive benefits of SF, highlighting the critical role of the BDNF-TrkB pathway in counteracting LPS-induced cognitive and memory dysfunction [141].

While myeloperoxidase (MPO) is primarily involved in antimicrobial defense, its activity is closely linked to inflammasome-mediated inflammatory responses including those in brain ischemia injury, and it can also be used to estimate leukocyte recruitment to tissues [142]. From rat models of liver injury induced by intestinal ischemia/reperfusion, we have seen that the administration of SF markedly reduced MPO activity with a concomitant elevation of GSH levels, liver Nrf2 and HO-1 expression, and increased GPX activity [143]. Similarly, SF has been suggested as a potential candidate for adjuvant therapy against cerebral ischemia–reperfusion injury, and experimental results showed SF to improve outcomes and slow cerebral ischemic/reperfusion injury via inhibition of NLRP3 inflammasomes and reduced MPO levels, reflecting neutrophil infiltration in the ischemic cerebral cortex [144,145].

SF’s powerful inflammation-modulating properties also suggest potential benefits for mood regulation and depression prevention. This connection is supported by evidence that unregulated neuroinflammation contributes to the development of depression and depressive symptoms, with effects manifesting as early as adolescence. Notably, Nrf2 knockout mice exhibit higher levels of inflammation and a depressive-like phenotype [146]. Using a social defeat stress model that mimics the effects of bullying and intimidation on depressive symptoms, the researchers found that a 3-week consumption of a GR-fortified diet (at a concentration of 0.1%) not only prevented the development of depressive behaviors but also mitigated reductions in Nrf2 and BDNF levels typically observed in untreated animals [146]. These findings underscore SF’s potential to counteract both stress-induced neurodegenerative changes and depressive symptoms, highlighting its promise as a multifaceted neuroprotective agent.

### 2.6. The Gut–Brain Axis

Strictly speaking, the phrase “the gut–brain axis” refers to the bidirectional communication between the gut and the brain. This is an area of very active research, and it is an outgrowth of the explosion in knowledge about the gut microbiome that has occurred since about 2005. There are now compelling studies suggesting that SF could have an impact on brain function through gut-related mechanisms. A few key areas of interest upon which we expand below include the fact that SF modulates microbiome composition, it reduces inflammation and enhances gut barrier integrity, and it may directly modulate the gut–brain signaling (e.g., vagus nerve).

#### 2.6.1. Gut Microbiota Modulation

SF can directly influence composition of the gut microbiota, which is now well-established as playing a critical role in the gut–brain axis [147]. In animal studies, SF affects the diversity and composition of gut microbiota, which could, in turn, influence brain function [148]. The modulation of gut bacteria might help in reducing gut-derived inflammation, which can affect the brain and contribute to cognitive decline. The effect of SF on the gut microbiome may also affect the production of short-chain fatty acids (SCFA) like butyrate, which have neuroprotective effects [149,150]. These SCFAs can promote the health of the blood–brain barrier, reduce neuroinflammation, and thus support brain function. These observations are complemented by a series of human clinical studies in which metabolism of SF was shown to be influenced by the gut microbiome composition [151], and consumption of cruciferous vegetables or broccoli altered the composition of the gut microbiome and shifted its physicochemical environment [152,153,154]. Explorations of the interplay between cruciferous vegetables and the gut microbiome have been continued by Bouranis and colleagues via multi-omics and other approaches [155,156,157].

#### 2.6.2. Reduction in Gut Inflammation

SF improves gut health by reducing intestinal inflammation and increasing the integrity of the gut barrier [158]. A compromised gut barrier (often referred to as “leaky gut”) can allow harmful substances, undigested food particles, and microorganisms, to enter the bloodstream, triggering systemic inflammation that can negatively affect systemic health in general, and brain health specifically. By enhancing gut barrier function, SF can reduce systemic inflammation and potentially protect the brain from neuroinflammation. Sulforaphane’s well-known anti-inflammatory properties, through up-regulation of Nrf2 and inhibition of the NF-кB pathway, can help reduce inflammation not only in the brain but also in the gut and elsewhere [159]. This is important to the extent that this lowers levels of circulating inflammatory cytokines that can cross the BBB and subsequently impair cognitive function.

#### 2.6.3. Gut–Brain Signaling Pathways

There are studies suggesting that compounds like SF could influence brain function via the vagus nerve, a key connection in the gut–brain axis [160]. The vagus nerve is involved in transmitting signals from the gut to the brain, and any notable progress in gut health (e.g., reduced inflammation or improvements in the composition of the gut microbiota) could lead to beneficial effects on brain function. While more research is needed to clarify this in the context of SF, it is a highly plausible mechanism. A more direct connection to the gut–brain signaling pathways may be that the gut microbiota produces a variety of metabolites including neurotransmitters that can positively or negatively influence brain health. The impact of SF in balancing gut microbial activity and favoring “good” bacteria over harmful bacteria can potentially increase the production of neurochemicals like serotonin or dopamine, which play supportive roles in cognition, mood, and mental health.

#### 2.6.4. Impact of SF-Induced Microbiome Changes on Neurological Disorders

The relationship between the gut microbiota and neurodevelopmental conditions such as ASD as well as neurodegenerative diseases like Parkinson’s and Alzheimer’s has gained much attention in recent years. There is now compelling evidence, particularly with ASD, that changes in gut microbiota composition could influence disease development and progression. At least one clinical study on ASD has been initiated (NCT02909959), in which the effects of SF supplementation on the gut microbiome were to be ascertained, though the results have not yet been published.

### 2.7. Other Mechanisms

#### 2.7.1. Neurogenesis and Synaptic Plasticity

Neurogenesis, the process by which neural stem cells produce new neurons, is vital for learning and memory. However, this process becomes dysregulated in many neurodegenerative diseases and declines naturally during normal aging. SF enhanced neurogenesis in a triple knockout transgenic mouse Alzheimer’s model [161] by increasing the neuronal expression of BDNF, which supports neuron generation. The Wnt/β-catenin pathway comprises a family of proteins that play critical roles in many tissues, including but not limited to embryonic development and adult tissue homeostasis. In neural stem cells, SF upregulates Wnt signaling, promoting their proliferation and differentiation into functional neurons [162].

SF also possesses a remarkable ability to enhance neuronal plasticity. Research by Yao and colleagues has shown that SF increases nerve growth factor-induced neurite outgrowth in a dose-dependent manner [146]. Improvements in synaptic plasticity are directly related to enhancements in learning and memory [163]. In addition to inhibiting apoptosis, Bcl-xL regulates mitochondrial bioenergetic efficiency, enhanced synaptic plasticity, synaptic vesicle recycling, neurite growth and healthy neuronal activity [164]. The ability of SF to consistently upregulate Bcl-xL, a protein known to prevent the mitochondrial release of pro-apoptotic factors as further described in Section 2.7.3, is another neuroprotective mechanism that can be targeted by SF—leading to promotion of normal synapse function, neuronal activity, and brain plasticity.

#### 2.7.2. The Heat Shock Response (HSR)

The neuroprotective effects of SF have been extensively studied in relation to the heat shock response (HSR), a crucial cellular defense mechanism against stress (Figure 5C). The HSR is a complex and evolutionarily conserved cytoprotective process that protects cells by activating the transcription of genes encoding molecular chaperones, proteases, and other essential proteins for cellular repair and recovery. Under stress conditions, heat shock proteins (HSPs), such as HSP70 and HSP27, bind to misfolded proteins, helping to refold them, prevent aggregation, and regulate their degradation. Other HSPs, such as HSP90, play an integral role in signal transduction, immunity, and apoptosis, such that their modulation could be directly beneficial for treating various human diseases [21]. Evidence suggests that cells are more vulnerable to damage when HSPs are suppressed. Since the inducible form of HSP70 is typically undetectable under normal conditions, its expression is often regarded as a diagnostic marker for stress. In the nervous system, HSP70 overexpression has been shown to protect against insults like heat shock and metabolic stresses through multiple mechanisms, such as its chaperone, anti-inflammatory, and anti-apoptotic functions [165,166], as well as protecting from experimental stroke in a mouse model [167].

The extensive effects of SF on HSPs include the following: (a) It is a very efficient inducer of HSP32 (also known as heme oxygenase 1 or HO-1). (b) It is a powerful activator of the heat-shock transcription factor 1 (HSF1)-mediated response in cultured cells, and it elevates proteasomal activity through the upregulation of HSP27. (c) It enhances the expression of HSP70, HSP90, and HSP40 in normal human fibroblasts. This SF-induced activation of the so-called “stress proteome” may involve at least two widely studied and interrelated cellular signaling pathways: the regulation of the Keap1/Nrf2/ARE cytoprotective genes, and the HSP/proteasomal pathway, both of which play a role in protecting against a wide variety of disturbances of cellular function [21].

#### 2.7.3. Apoptosis

Unregulated apoptosis or programmed cell death of neural cells is a common feature of age-related cognitive decline and neurodegenerative conditions. It is also another mechanism that is regulated in part through the Nrf2 pathway [31]. The Bcl-2 family of anti-apoptotic proteins, which can be upregulated in neurons by SF, have been identified as additional downstream gene products of Nrf2 regulation [22]. Interestingly, one of these proteins, Bcl-xL, has also been found to regulate mitochondrial bioenergetic efficiency, synaptic transmitter release, synaptic vesicle recycling and neurite growth, suggesting additional protective mechanisms for anti-apoptotic proteins [164]. For example, SF protected cultured neurons against oxygen and glucose deprivation/reoxygenation-induced injury and resultant apoptosis through increased expression of Bcl-2 [168]. Similarly, SF pretreatment protected neural cells against Aβ-fragment cytotoxicity and apoptosis in part through upregulation of Bcl-2 [169]. In the same experiments, it also increased expression of GCL, NQO1, and HO-1 in parallel with reductions in 4-HNE and protein carbonyls, two key markers of protein oxidation.

#### 2.7.4. Autophagy

Autophagy, loosely defined, embraces the cellular processes responsible for the targeted degradation and removal of damaged cells, proteins, and organelles, including damaged mitochondria (mitophagy). One of the consequences of the high metabolic flux (i.e., mitochondrial activity) in the brain is an increase in protein oxidation. This is exacerbated by neuroinflammation such that the accumulation and aggregation of cytotoxic damaged proteins is a hallmark of most neurodegenerative diseases. Autophagy plays an important role in mitochondrial quality control, and upregulation of autophagy by SF is yet another strategy to prevent age-related cognitive damage and the development of neurodegenerative diseases [170,171]. Part of the work of autophagy is performed by proteasomes, and activation of proteasomes for removal of damaged proteins is one of many beneficial consequences of delivering SF to cells as SF has been shown to increase damaged protein clearance via Nrf2-modulated regulation of the proteasome system [31].

#### 2.7.5. Epigenetic Effects

Epigenetics refers to heritable changes in gene expression that are made without altering the DNA sequence, often mediated by processes such as histone modification (e.g., with a histone deacetylase, HDAC), DNA methylation, and microRNA regulation. Unfortunately, epigenetic mechanisms of SF in the brain continues to be an understudied area. However, modulating epigenetic processes may have profound implications for brain health and cognition, particularly in conditions involving neurodegeneration and cognitive decline [172].

One of SF’s primary epigenetic actions is the inhibition of histone deacetylases (HDACs), enzymes that remove acetyl groups from histones, leading to a condensed chromatin structure and reduced gene expression [173]. In part, this may be accomplished by SF because when linked to N-acetylcysteine it can bind to the active site of the HDAC using a Zn atom [174]. Dashwood and colleagues first showed that SF was an inhibitor of HDAC that might be useful in cancer prevention [175]. Then, in 2009 they demonstrated that SF-modulated HDAC inhibition directly results in increased expression of Nrf2 target genes, which protect against oxidative stress and inflammation [176]. Although this group focused their study on SF’s role in cancer prevention, both oxidative stress and inflammation are also critical factors in cognitive health. By inhibiting HDACs, SF promotes a more relaxed chromatin state, typically enhancing the transcription of genes involved in antioxidant defenses, neuroprotection, and synaptic plasticity. For example, the study that demonstrated SF’s effect on neural plasticity using the triple-transgenic mouse model of Alzheimer’s disease [177], showed that SF regulated BDNF expression specifically via HDAC inhibition.

Furthermore, SF can modulate DNA methylation patterns [173]. SF, as well as its metabolites, decreases gene expression of DNA methyltransferases (DNMT) 1 and DNMT3b. As a result, methylation of promoter regions of critically important cell cycle regulatory genes, including but not limited to cyclin D2 and c-Myc, was reduced [178]. Yang et al. reported that SF can alter methylation of promoters for genes involved in neural plasticity and inflammation, thereby modulating their expression [179]. This property is particularly relevant in age-related cognitive decline and neurodegenerative diseases, where dysregulated methylation patterns are highly associated with disease progression. It has been hypothesized that by restoring balanced methylation, SF may help to preserve neuronal function and improve memory.

And finally, SF impacts microRNA (miRNA) profiles, which regulate post-transcriptional gene expression. Zhao et al. highlighted SF’s ability to modulate miRNAs linked to neuroinflammation and oxidative stress, further supporting its role in maintaining brain health and cognitive function [180].

## 3. Disorders

This section is guided in part by the magnitude of published experimental and clinical work on the conditions, and in part on the broader public health interest in these topics. In a review of this nature, not all conditions can be adequately discussed in which an effect of SF has been: (a) speculated upon, (b) demonstrated, (c) assumed, based on animal models, or (d) shown in the clinic. In Table 3, we present a list of the disorders considered, and a complete listing of studies appearing on clinicaltrials.gov is presented as Supplementary Table S1.

A great many animal (primarily rodent) studies have been performed by treatment with either GR or SF (dietary, intraperitoneal, or topical), and some of those have outcomes that are difficult to relate to specific human conditions. However, certain studies could be correlated with a number of brain-health related conditions: for example, those related to behavioral abnormalities in offspring after maternal (perinatal) immune activation [181], and those related to Nrf2-related anti-aging strategies [182]. Those will not be further discussed, but their existence should be acknowledged.

### 3.1. Mood, Cognitive, and Psychiatric Disorders (Affecting Mental Health and Cognition)

#### 3.1.1. Mood, Depression, and Anxiety Disorders

Mood disorders, including depression and anxiety, share underlying mechanisms involving immune dysregulation, oxidative stress, and dysfunction of the hypothalamic–pituitary–adrenal (HPA) axis [183]. SF has demonstrated promising antidepressant and anxiolytic effects in various animal models, with emerging clinical evidence supporting its potential in humans.

*Mood Disorders.* SF reduces pro-inflammatory cytokines (IL-6, TNF-α), increases anti-inflammatory cytokines such as IL-10, and enhances microglial activity, suggesting a role in mitigating neuroinflammation-related mood disorders [184,185]. Additionally, Wu et al. have suggested that SF may modulate the GABAergic system, which plays a crucial role in inhibitory neurotransmission and anxiety regulation [184].

*Depression.* In multiple animal models, SF has reversed depressive-like behaviors induced by both acute and chronic stress. In chronically stressed mice, SF administration reduced corticosterone, adrenocorticotropic hormone, IL-6, and TNF-α, effectively mitigating stress-induced neuroinflammation [184]. In a separate study, juvenile and adolescent mice treated with 0.1% dietary GR (the biogenic precursor of SF), were protected from developing a depression-like phenotype in adulthood despite chronic stress exposure. Moreover, Nrf2 knockout mice were more sensitive to chronic stress than their wild-type counterparts, highlighting the critical role of the Nrf2 cytoprotective response in mitigating depression [146]. In an LPS-induced depression model, which mimics inflammation-related depressive symptoms, SF treatment exhibited prophylactic effects by reducing TNF-α levels, enhanced microglial activity, increased anti-inflammatory IL-10 levels, and improved depressive behaviors compared to untreated mice [185]. These findings suggest SF’s potential to counteract both stress-induced and inflammation-driven depressive states. A 12-week, 90 subject clinical study of the effects of SF on depression is ongoing in Changsha, China, at the time of this writing (NCT04246905). 

*Anxiety.* SF has also demonstrated anxiolytic effects, particularly in models where anxiety is linked to chronic pain and neurodegenerative conditions [184,186]. In neuropathic pain models, SF administration reduced anxiety-like behaviors, suggesting its potential for alleviating anxiety associated with persistent pain conditions [187]. In Alzheimer’s Disease (AD) models, SF-treated mice spent more time in the open arms of the elevated plus maze and in the center of an open field test, indicating reduced anxiety compared to untreated AD mice [187] and suggesting SF’s potential in mitigating anxiety symptoms related to neurodegenerative disorders.

While preclinical evidence is strong, human studies on SF’s effects on anxiety remain limited. However, a randomized, double-blind, placebo-controlled clinical trial found that SF improved depressive symptoms in patients with a history of cardiac interventions, suggesting potential benefits for anxiety as well due to their frequent comorbidity [186].

#### 3.1.2. Autism Spectrum Disorder (ASD)

Autism is one of the most common neurodevelopmental disorders in the United States. It has most recently been estimated to affect 1 out of 36 children aged 8 years old [188]. Yet despite decades of research and advances in our knowledge of the etiologies of ASD, treatments and biomarkers for ASD remain limited [189]. To date, the primary diagnosis of ASD still relies on observational tools that are subjective in nature. Genetic and metabolic studies may provide additional clues to specific etiologies or treatments, but there are no generally accepted biological markers for commonly affected cellular pathways in ASD.

Studies over the last two decades have shown that physiological and metabolic abnormalities, such as immune dysregulation/neuro-inflammation, redox imbalance/oxidative stress, mitochondrial dysfunction, environmental toxicant exposures and gut dysbiosis, can all be important aspects of the pathophysiology of ASD [164]. Closer examination of the biological markers of pathways associated with ASD could be informative regarding its pathophysiology, and might be useful for early identification, prognosis, and treatment. Most importantly, they might guide treatment strategies and enable clinicians to monitor treatment responses. Since ASD is multi-factorial, and multiple genes have been implicated without specific drug targets, strategies using multi-functional phytochemicals are highly attractive [190,191,192,193].

SF, prime among these phytochemical options, has now been the subject of at least eight clinical studies, many of them now completed, some of them already published, and all of the published outcomes except one [194] showed beneficial effects [71,80,195,196,197,198,199,200]. The first of these trials [197] was an 18-week double-blind randomized controlled trial (RCT) with 45 adolescent and young adult males. SF treatment resulted in improvements in social interaction, aberrant behavior and verbal communication based on the Aberrant Behavior Checklist (ABC) and Social Responsiveness Scale-2 (SRS-2). Furthermore, the SF arm showed improvements compared to the placebo arm: in biomarkers of GSH redox status, mitochondrial respiration, inflammatory markers, and heat shock proteins. After completing the original intervention, the progress of a subset of the study participants was followed for up to 3 additional years; many of them continued to take SF supplements, and a number of them reported continued improvement in the study participants’ ASD symptoms [196]. Subsequently, a 12-week open label study, which enrolled 15 children with ASD measured similar primary outcomes by ABC and SRS-2 [195]. There were significant improvements in some subscales, though not as robust as in the Singh (2014) study [197]. In this study, though, urinary metabolomics identified 77 urinary metabolites, including seven different sphingomyelins, which were correlated with symptom improvement following daily SF consumption. Since these sphingolipids are so important in membranes and in the myelin sheaths of nerve axons, we [195] posited that their potential connection to ASD pathophysiology ought to be more aggressively investigated. This has now been followed up upon and indeed there were either significant increases or decreases in plasma sphingomyelin associated with symptom severity [201]. Further to that, a study giving SF or placebo to human subjects with ASD was just published, available to the authors in abstract form only, which states that “The molecular mechanism of SF in improving ASD related clinical symptoms may be related to cell membrane phospholipid metabolism. Sphingomyelin (d35∶1) and taurine may be possible predictors on the efficacy of sulforaphane in the treatment of ASD” [202].

A 12-week, randomized, double-blind, placebo-controlled, multicenter SF intervention trial was conducted in China with children (aged 3–15 years) with ASD in which 135 children were randomized to either SF or placebo treatments [198]. Clinical outcomes included SRS, Clinical Global Impression Scale (CGI), Repetitive Behavior Scale (RBS-R), ABC, and OSU Autism Rating Scale (OARS-4). There were significant (at least 30%) improvements in participants in the SF group by the 12th week of treatment when rating scales that required clinician rating were used, but puzzlingly, no significant changes between SF and placebo groups in caregiver-rated scales.

A clinical trial published in 2021 [71], was a direct follow-up to the first study on SF and ASD, and was conducted by the same team that performed the initial trial [197]. In the initial study, biomarkers were not able to be monitored, so the strength of these results relied solely on ASD evaluation scales (ABC and SRS) scored by caregivers and by the highly trained medical staff conducting the study. In the follow-up study these metrics were used, but a variety of blood-based biomarkers were quantified as well. Many of these biomarkers exhibited positive changes with SF treatment as has been reported [71,80]. SF decreased oxidative stress by reducing the GSH/GSSG ratio (ratio of reduced to oxidized glutathione) compared to placebo, and it increased certain detoxification and antioxidant markers (like NQO1) and decreased inflammatory markers (IL-6, IL-1β, TNF-α) [80]. Furthermore, SF treatment showed promising effects on mitochondrial function in that there was improvement in mitochondrial parameters such as ATP-Linked Respiration and Proton Leak Respiration in the SF group compared to the placebo group. Notably, these improvements correlated with positive changes in behavioral ratings on the ABC scale [71]. The link between mitochondrial function and ASD symptoms may be explained through the concept of ‘oxoinflammation’, the interplay between oxidative stress and inflammation [203,204]. This connection is particularly relevant in ASD, where decreased Nrf2 expression has been observed [205]. Nrf2 is a key regulator of oxoinflammation [206,207], and SF is known to be a potent inducer of Nrf2 signaling [208,209]. Interestingly, SF treatment had differential effects on mitochondria respiratory capacity in children with ASD, depending on their clinical subgroup. Children who had experienced developmental regression showed decreased levels of respiratory capacity with SF treatment, while those without regression exhibited increased respiratory capacity [71]. This finding underscores the importance of identifying and considering clinical subgroups in future ASD clinical trials involving SF or similar interventions.

Two additional clinical SF intervention studies (NCT02677051 and NCT04805957) remain active and one (NCT 0209959) has been completed but has yet to publish results. The protocol for this completed trial at UNC, Chapel Hill, also calls for collection of stool samples for microbiome analysis. Microbiome effects on the symptoms of ASD are an area of major interest. One particularly noteworthy example comes from Arizona State University, in which the delivery of a large bolus of commensal microbes from a healthy donor to 18 participants with ASD had beneficial effects on ASD symptoms, gut microbiota, and GI symptoms for more than 2 years following treatment [210]. Expectations in the ASD community are high that exciting findings will be forthcoming. In a further published example, following 12 weeks of daily administration of SF to both ASD-subjects and neurotypical control children (aged 4–7 years), an abundance of 25 microbial taxa correlated with improvements in ASD symptoms and associations of two of these taxa correlated with those identified in an ASD-like rat model and presented in the same paper [200].

#### 3.1.3. Schizophrenia

Schizophrenia is a progressive neurodevelopmental disorder, which disrupts an individual’s ability to think clearly, regulate emotions, make decisions, and maintain relationships. Like ASD, it is a complex and variable condition with a significant genetic component. Schizophrenia is typically managed with high doses of antipsychotic medications. Symptoms often emerge during a prodromal phase before full onset, which has prompted interest in early interventions such as dietary intake of potent indirect antioxidants like SF.

Mouse models have been used in which (a) a GR-rich diet protected the offspring of mice subjected to maternal immune activation from cognitive defects—a protective effect that persisted into adulthood [211]; (b) the cognitive deficits induced in juvenile males by repeated administration of the *N*-methyl-D-aspartate (NMDA) receptor antagonist phencyclidine (PCP) were attenuated by both SF and GR [113].

Findings from two small human trials have provided further insights. In a small open-phase trial, 8 weeks of SF treatment led to improved accuracy in the One Card Learning Task among individuals with schizophrenia, although no other significant changes were observed [212]. In healthy human volunteers, 7 days of daily SF ingestion increased glutathione (GSH), the body’s most abundant antioxidant, in the blood. This increase correlated with elevated GSH levels at specific brain regions, as measured by 7-Tesla magnetic resonance spectroscopy imaging [70]. The latter finding is particularly important because, for the first time, GSH levels in specific areas of the brain associated with dysfunction in schizophrenia were shown to be upregulated by SF from broccoli extracts and these increases in GSH were mirrored in systemic circulation.

Biomarker-based clinical trials in China and the United States have now investigated the effects of SF on schizophrenia in larger clinical studies. In a 4-site, 172-patient, 22-week RCT of patients with first episode schizophrenia in China, there was no significant improvement in primary outcomes (MATRICS, MCCB, and PANSS scores), in patients given SF supplements [213]. There were, however, improvements in a number of domains within the MATRICS test suggesting to the investigators that SF may have utility as an adjunct for some types of cognitive dysfunctions in schizophrenia.

A 16-week single-blind placebo-controlled intervention (*n* = 58) of schizophrenia outpatients, conducted at Sheppard-Pratt Psychiatric Hospital in Baltimore, MD measured symptom severity (PANSS), cognitive functioning (MATRICS), and markers of inflammation. Following 16 weeks of daily oral SF there were no changes in these primary endpoints [214]. However, there were significant and dose-dependent increases in working memory by level of SF and its metabolites measured in the urine of these subjects [215].

An ongoing study in Shanghai (NCT03932136) seeks to recruit 300 schizophrenia patients at risk for psychosis and perform a 1 year dietary SF intervention with an additional 1 year follow-up. A related, smaller study (NCT04521868) has just been published in which a total of 53 subjects were randomized to a SF arm and 24 subjects to a placebo arm for 12 weeks of treatment and 24 weeks of follow-up, with the objective of assessing whether SF could reduce the negative symptoms of the antipsychotic medications that all subjects were taking [216]. Patients given SF for 24 weeks had a significantly greater reduction in Positive and Negative Syndrome Scale (PANSS) negative symptom scores and PANSS negative factor scores than placebo-treated subjects, and this reduction was not affected by changes in scores for depression or cognitive factors.

And in yet another approach to schizophrenia, mutations in the astrocyte-enriched enzyme aldehyde dehydrogenase 7a1 (ALDH7A1) cause a neonatal epilepsy accompanied by treatment-resistant, inter-ictal neuropsychiatric symptoms. Faust and collaborators created ALDH7A1 knockout mice and showed that constitutive global ALDH7A1 depletion increases chemoconvulsant sensitivity and altered mood-associated behaviors. However, this astrocyte-specific ALDH7A1 depletion only affected mood-associated behaviors. SF rescued both the physiological and behavioral changes by targeting astrocytic redox imbalance in ALDH7A1 knockout mice, implicating astrocyte redox changes in creating cortical excitatory–inhibitory imbalance and mood alteration [217].

Though at present not as exciting as, for example, results with ASD, these results suggest that SF, through its antioxidant and neuroprotective properties, may promise as a supplementary intervention for managing schizophrenia and mitigating cognitive impairments associated with the disorder.

#### 3.1.4. Diabetes-Related Cognitive Decline

Strong evidence suggests a pathophysiologic link between type 2 diabetes mellitus and cognitive dysfunction/decline including an increased risk of developing Alzheimer’s disease [218]. Defects in insulin signaling, neuroinflammation, and mitochondrial metabolism have been invoked as potential causative factors. The ability of SF to attenuate diabetic complications and related cognitive decline has been evaluated. In a streptozotocin diabetic rat model, animals treated daily for 14 d with 25 mg/kg SF exhibited a reduced decline in memory and learning, apoptosis of their hippocampal neurons, and abnormal expression of critical signaling molecules (e.g., caspase-3 and induced myeloid leukemia cell differentiation protein (MCL-1)) [101]. Nrf2 signaling deficits in diabetic mice have been linked to BBB disruption [219]. Human clinical studies have demonstrated positive effects of SF on the symptoms of T2DM, but they have not yet examined associated cognitive disfunction [220,221].

#### 3.1.5. Cognitive Function and Memory Impairment

SF has shown promising effects in improving cognitive function and mitigating memory impairment. These benefits are attributed to SF’s ability to combat oxidative stress, inflammation, and neurodegeneration, which are common pathological mechanisms underlying cognitive decline in conditions such as aging, Alzheimer’s disease, and traumatic brain injury. SF activates the Keap1/Nrf2 pathway, leading to the upregulation of antioxidant and detoxifying enzymes that protect against neuronal damage and support cognitive health. This has been underscored using a phencyclidine-induced cognitive deficit mouse model [113]. More recently it was demonstrated that SF increases mitochondrial biogenesis-related gene expression in the hippocampus of mice and suppresses age-related cognitive decline [84].

Preclinical studies with rats have demonstrated SF’s potential to enhance cognitive performance after traumatic brain injury. Dash and colleagues reported that if SF was administered at 1 h post-injury, but not at 6 h, it improved cognitive function, spatial learning and memory (assessed on day-14 post-injury), and reduced working memory dysfunction (assessed starting at day 25 post-injury) [222]. This improvement was linked to SF’s ability to protect neurons and other neurovascular unit components from oxidative damage based on anti-4-hydroxy-nonenal reactivity in the hippocampus of treated vs. control animals. Similarly, Kim et al. [161] found that SF ameliorated memory deficits in a mouse model of Alzheimer’s disease by reducing amyloid-beta deposition, oxidative stress, and neuroinflammation. These findings highlight SF’s dual action in addressing both structural and functional aspects of memory impairment.

Moreover, SF has been shown to modulate synaptic plasticity, a key mechanism for learning and memory. The effects of SF have been examined using a scopolamine-induced memory impairment mouse model—scopolamine is used to generate amnesia for the screening of antiamnesic drugs. One group found that SF exerted neuroprotective effects by enhancing the cholinergic system. Specifically, SF increased acetylcholine (ACh) and choline acetyltransferase levels in the hippocampus and cerebral cortex while reducing acetylcholinesterase (AChE) activity. This dual action helped preserve cognitive function and prevent the progression of neurodegeneration [99]. Others then used a similar model to demonstrate that SF treatment enhanced long-term potentiation in the hippocampus, a critical brain region for memory formation. They found that SF increased total field excitatory postsynaptic potential (fEPSP) in a dose-dependent manner after high frequency stimulation and that SF attenuated scopolamine-induced interference of the fEPSP in the hippocampus as well as preserving synaptic integrity and reducing the neuroinflammatory markers TNF-α and IL-1β [223].

In a randomized controlled trial (RCT) combining brain training with supplementary SF, SF improved cognitive performance in healthy older adults [224]. In the SF arm of the intervention, participants had significant improvement in processing speed, and working memory performance, compared to the placebo intake group, as measured by subsets of the Wechsler Adult Intelligence Scale 3rd (WAIS-III). In a separate RCT in which SF was delivered daily for 12 weeks, the same research group documented improved processing speed as well as mood improvement compared to the placebo arm, using both WAIS-III and Profile of Mood State Second Edition (POMS2) [225].

### 3.2. Neurodegenerative Diseases (Progressive Degeneration of Neurons)

#### 3.2.1. Alzheimer’s Disease (AD)

Alzheimer’s disease, the leading neurodegenerative disorder worldwide, has been estimated to affect over 416 million people (combined AD dementia, prodromal AD, and preclinical AD), or 1/5 of all persons aged 50 and above, worldwide [226]. Studies in animal models suggest that ITCs, including SF, may help prevent hallmark features of AD, such as tau protein tangles and amyloid-beta (Aβ) plaques, while also slowing cognitive decline. Research on SF-treated mouse cortical neurons and a triple-transgenic AD in vivo mouse model revealed increased expression of BDNF, potentially linked to SF’s ability to epigenetically enhance neuronal BDNF expression through histone acetylation and HDAC inhibition [177].

In the same AD mouse model, oral SF administration reduced Aβ and tau protein levels by upregulating heat shock protein HSP70 and its co-chaperone CHIP (carboxy-terminal Hsp70-interacting protein). These molecular changes translated to improved memory performance in object recognition and contextual fear conditioning tests [227]. Additional rodent studies reinforced SF’s neuroprotective effects. In one study, SF treatment improved spatial cognition, increased locomotor activity, and reduced Aβ plaque formation in the hippocampus and cerebral cortex [228]. Another study found that SF reduced the expression of HDACs1, 2, and 3, upregulated p75 neurotrophin receptor, and reduced Aβ plaque burden in the cerebral cortex of AD model mice [229]. Transgenic AD mice treated with SF also showed fewer Aβ aggregates and delayed cognitive decline [230]. Moreover, postnatal SF administration in mice promoted mechanisms involved in spatial learning and memory consolidation, showed that overcoming proteasome inhibition played an important role in hippocampal synaptic plasticity during the early postnatal period. Due to the involvement of decreased proteosomal activity in AD disease, these investigators posit that SF may have potential as a therapeutic strategy for this condition [231].

Some of the SF benefits in AD are attributed to the activation of pro-autophagy pathways, which facilitate the breakdown of abnormal protein aggregates. SF supplementation also decreases oxidative stress caused by protein aggregation, a key factor in AD pathology. In toxin-induced AD mouse models, SF helps preserve cholinergic neurons in the hippocampus and medial septal regions [232]. Additionally, it protects cultured neural cells from the toxicity of methylglyoxal, a precursor to AGEs linked to AD [122]. SF further reduces neural cell death triggered by Aβ exposure by activating proteasomes [227,231,233], and reverses Aβ oligomer induced depressive symptoms in a mouse model [234].

Administration of SF in various doses has demonstrated potential in improving cognitive dysfunction in AD animal models, as reported by [161]. While it showed limited ability to completely prevent Aβ aggregation, the Kim group’s study highlighted the overall ability of SF to mitigate cognitive decline by protecting against amyloid-related brain damage. Subsequent research on neurodegenerative disease models confirmed SF’s therapeutic promise. For instance, oral SF administration in an AD-like mouse model reduced cholinergic neuron loss and improved cognitive deficits caused by aluminum and D-galactose exposure [232]. Additionally, the same researchers later treated 4-month-old AD model mice by gavage daily for 5 months, with 25 mg/kg SF, which significantly reduced Aβ plaque levels in the hippocampus and cerebral cortex, improved cognition, and was shown to reduce HDAC 1 and 3 levels, suggesting its potential to counteract Aβ-induced cytotoxicity in AD models [232].

#### 3.2.2. Parkinson’s Disease (PD)

Parkinson’s disease is the second most common progressive neurodegenerative disorder and is characterized by the loss of dopaminergic neurons in the substantia nigra and the accumulation of α-synuclein aggregates, leading to motor and non-motor symptoms. Oxidative stress, mitochondrial dysfunction, and neuroinflammation are key contributors to PD pathology, making SF an obvious candidate for therapeutic intervention due to its ability to target these mechanisms.

A variety of neurotoxins are used in rodent PD models. With these models, SF has been shown to protect dopaminergic neurons by reducing oxidative stress and inflammation. For instance, it was reported that SF treatment via diets amended with 0.1% glucoraphanin for 28 days improved motor function and reduced dopaminergic neuron loss in a mouse model of PD induced by methyl-4-phenyl-1,2,3,6-tetrahydropyridine (MPTP), a toxin that selectively targets dopaminergic neurons [114]. These effects were associated with increased Nrf2 activation and the upregulation of downstream antioxidant enzymes like HO-1 and NQO1. Similarly, dietary glucoraphanin prevented the reduction in dopamine transporter in the striatum of mice following administration of MPTP [114]. Additionally, SF has been found to mitigate neuroinflammation by inhibiting the activation of microglia and astrocytes, key mediators of inflammatory responses in the PD brain. In a 6-hydroxydopamine mouse model in which SF (20 µmol/kg) were given i.p., it reduced pro-inflammatory cytokine levels and protected against dopaminergic neuron degeneration compared to control mice [235,236]. SF also demonstrated potential to reduce α-synuclein aggregation, as shown in in vitro studies, further highlighting its multifaceted neuroprotective actions [237]. Knockout (KO) models in mice with inhibited mitochondrial respiration leave rodents at risk of dopamine-induced neurodegeneration. When SF was supplied to cultured midbrain neurons and astrocytes from KO mice, dopamine-induced cell death was reduced, and mitochondrial membrane potential was restored [126,238]. The potent toxin rotenone induces locomotor activity deficiency and dopaminergic neuronal loss, which is reversed by SF treatment and associated Nrf2-dependent reductions in oxidative stress, restoration of normal autophagy, and mTOR-dependent inhibition of neuronal apoptosis [106].

A recent 8-subject clinical experiment has utilized SF and reported results as follows: In this study of persons with Parkinson’s, SF “induced powerful and concurrent attenuation of a diverse group of Parkinson’s non-motor symptoms, including fatigue, constipation and urinary urgency. Motor symptoms were strictly unaffected. This observation indicates that oxidative stress may be a common factor contributing to non-motor symptoms involving sites in the CNS and peripheral organs”. It goes on to conclude that “We tentatively interpret the results in terms of a hypothetical model for Parkinson’s Syndrome which we describe as a multisystem redox disorder with reservoirs of the disease in peripheral organs as well as in the brain.” [239]. While exceedingly intriguing, there are many aspects of this preliminary study which need to be replicated in a highly controlled manner. Nevertheless, it provides hope and a new perspective from which to view the effects of dietary (e.g., broccoli) interventions. 

There is an ongoing 24-week, 100-person placebo-controlled study of SF’s effect on PD (NCT05084365).

#### 3.2.3. Huntington’s Disease

Huntington’s disease is a hereditary neurodegenerative disorder with an autosomal dominant inheritance pattern. Symptoms typically present in adulthood with motor impairments, psychiatric symptoms, and progressive cognitive decline. Mitochondrial dysfunction plays a key role in the pathogenesis of Huntington’s disease, as it does with other neurological disorders like ASD and PD, and pharmacologic interventions are limited [240]. In a rodent model of Huntington’s disease, SF demonstrated neuroprotective effects by counteracting damage induced by quinolinic acid. These protective effects were achieved through the restoration of mitochondrial function, leading to the suggestion that SF may have potential to ameliorate Huntington’s symptoms [241]. Both in vitro and in vivo work on the ubiquitin proteasome system, which is impaired in Huntington’s disease, can be stimulated by SF, leading to reduced accumulation of mutant huntingtin (mHtt) protein aggregates, the accumulation of which is a main cause of Huntington’s disease [242]. Very recently published work with cultured transdermal fibroblasts showed that SF-induced autophagy and/or translation block can also protect cells against an accumulation of these mHtt aggregates [243].

### 3.3. Brain Injuries and Acute Events (Brain Injuries or Events That Disrupt Normal Brain Function)

#### 3.3.1. Traumatic Brain Injury (TBI) and Spinal Cord Injury (SCI)

TBI is increasingly recognized as a major global cause of mortality, long-term disability, and cognitive impairment following head trauma. Oxidative stress and inflammation play key roles in its pathogenesis, and this has been addressed by a number of preclinical studies. For example, a post-injury SF treatment of rats improved learning capacity and performance in working memory tasks, hypothesized to arise from SF’s ability to shield neurons and other neurovascular unit cells from oxidative damage [222], which have previously been directly linked to Nrf2-driven gene transcription by this group [244,245]. They determined that when administered intraperitoneally, 5 mg/kg SF reduced neuronal death, contusion volume, and neurological dysfunction 7 days post-TBI, and those neuroprotective effects were diminished in Nrf2-knockout mice, underscoring the important role of the Nrf2 pathway in TBI [245]. Furthermore, extravasation of a tracer compound was reduced during the acute phase of TBI if SF was administered prior to injury [246]. And neuroprotective effects of SF were attenuated in Nrf2 knockout mice in yet another study [247]. At least two studies suggest that SF’s neuroprotection may stem from decreased BBB permeability, improved cell survival, and/or enhanced aquaporin-4 (AQP4) channel presence. The first was the previously mentioned study by Zhao and colleagues in which BBB permeability was modified when SF was given 6 h post-injury. These benefits were linked to the activation of Nrf2, as evidenced by elevated expression of Nrf2-regulated genes such as GST, HO-1, and GSx in the cortex and cerebral microvessels [245]. The second of these rodent models revealed that similarly administered SF preserved AQP4 channels, increasing AQP4 protein levels in the peri-lesion area at 24 h and 3 days post-TBI as determined by immunofluorescence and mRNA measurement [244]. Collectively, these studies highlight SF’s potential to counteract various pathophysiological effects of TBI, primarily through activation of the Nrf2 pathway.

In a rat SCI model, two doses of SF (10 or 50 mg/kg) were administered at 10 min and 72 h after contusion SCI. SF at the higher dose treatment resulted in both acute and long-term beneficial effects, including upregulation of the phase 2 antioxidant response at the injury site, decreased mRNA levels of inflammatory cytokines in the injured spinal cord, inactivation of urinary macrophage MIF tautomerase activity (which is thought to act by blocking its interaction with the CD74 receptor of macrophages), and by enhancing hindlimb locomotor function, and an increased number of serotonergic axons caudal to the lesion site [248]. Simultaneous parallel reports of the effectiveness of SF in mouse and rat models of SCI supported these findings [249,250]. These studies were conducted in a mouse model of compression SCI, in which SF (5 mg/kg i.p.) was administered 1 h after injury [250], and in a rat model of contusion SCI, in which SF (5 mg/kg i.p.) was administered 15 min after injury and once daily for 3 days thereafter [249]. Treatment with SF reduced NF-кB pathway activation and decreased levels of cytokine gene expression and protein levels of MMP-9, TNF-α, IL-6, and IL-1β in the spinal cord lesions at 12 and 24 h after SCI [249,250,251].

A 90 subject clinical trial has been registered (NCT04252261) in which the objective is to assess the efficacy of sulforaphane for improving cognitive function in patients with frontal brain damage [252].

#### 3.3.2. Ischemia/Reperfusion-Induced Brain Injury

SF has been extensively studied in animal models for its neuroprotective effects in ischemia/reperfusion (I/R)-induced brain injury. This condition, often occurring during stroke or cardiac arrest, results from the restoration of blood flow to previously ischemic brain tissue, triggering a cascade of oxidative stress, inflammation, and neuronal death. SF’s ability to activate the Nrf2 signaling pathway and associated antioxidant enzymes NQO1, HO-1, and GPx positions it as a promising therapeutic agent for mitigating these damaging effects [33]. For example, Yu et al. [144] demonstrated in a rat model of transient cerebral ischemia, and Zhao et al. [253] in a separate study, that pretreatment with SF significantly reduced infarct volume and improved neurological outcomes. These studies attributed these protective effects to enhanced Nrf2 activation and increased levels of antioxidant defenses in the brain. Beyond its antioxidative properties, SF also mitigates neuroinflammation, a critical factor in I/R injury. Zhao et al. [254] reported that SF treatment reduced microglial activation and suppressed the production of pro-inflammatory cytokines such as TNF-α and IL-6 in a mouse model of cerebral I/R injury.

#### 3.3.3. Stroke

While less common than ischemic strokes, hemorrhagic strokes, particularly intracerebral hemorrhages (ICH), carry a higher mortality rate due to complications such as intracranial hypertension and brain herniation caused by hematomas and edema. Ischemia and oxidative stress further contribute to brain injury following ICH. SF has shown potential as a neuroprotective agent against hemorrhagic strokes.

Yin and colleagues explored the effects of SF in a rat model of ICH and found that SF activated the Nrf2 signaling pathway, increased HO-1 expression, and reduced the severity of neurological dysfunction compared to the control group [255]. Similarly, Zhao and colleagues demonstrated that SF mitigated ICH damage by activating Nrf2, reducing oxidative stress, and decreasing brain edema. Their findings also indicated that Nrf2 deficiencies weakened the neuroprotective effects of SF [256].

Furthermore, SF’s activation of Nrf2 was identified as a key factor in enhancing hematoma clearance after ICH. By stimulating Nrf2 in microglia, SF increased antioxidative capacity, phagocytosis, and hematoma resolution [254,257]. Additionally, SF treatment alleviated cerebral vasospasm and early brain injury in a subarachnoid hemorrhage model through Nrf2 activation, which promoted the production of antioxidant and detoxifying enzymes [256].

#### 3.3.4. Alcohol-Related Disorders

Alcohol (ethanol) has manifold deleterious effects on the human body, the brain, and cognition—it manifests toxicities of all grades [258,259]. One of the mechanisms of toxicity results from the fact that the main metabolic route by which ethanol is detoxified is via a series of two enzymes. First it is converted to acetaldehyde by alcohol dehydrogenase (ADH), and then that acetaldehyde is converted to acetate by aldehyde dehydrogenase (ALDH) [260]. The latter is non-toxic, and the former is both mutagenic and carcinogenic and its presence can lead to a variety of symptoms including headache, tachycardia, nausea, and flushing of the skin, which can be used as an indicator of alcohol intolerance. About 40% of East Asians have a mutation in ALDH (ALDH2*2), which results in decreased or absent ALDH activity and accumulation of the toxic acetaldehyde. Work at Johns Hopkins has shown that not only is SF a powerful inducer of ALDH (likely through the Nrf2 pathway), but that administration of SF results in a dramatic reduction in blood acetaldehyde levels in a mouse model [261]. Furthermore, they demonstrated that alcohol-intolerant individuals with presumptive ALDH mutations, could be readily identified by a rapid and novel skin-test [262].

Fetal alcohol syndrome (FAS) is a condition brought about by prenatal alcohol exposure that results from neural crest cells being excessively targeted for apoptosis, which leads to a wide range of embryo and phenotypic characteristics including a number of mental handicaps such as hyperactivity, anxiety, short attention span and learning disabilities [263]. SF diminished neural crest cell apoptosis in mouse embryos exposed to ethanol by epigenetic mechanisms (inhibiting HDAC and increasing histone acetylation), suggesting that it might be able to play a role in the amelioration of some of the brain-related FAS symptoms [264].

### 3.4. Epilepsy and Seizure Disorders (Affecting Motor Control and Electrical Activity in the Brain)

Epilepsy is the fourth most common neurological condition in the U.S., affecting between 1.2 and 3.4 million people in the United States and approximately 46 million worldwide. About one-third of the reported epilepsy cases are drug-resistant [265,266]. Epileptogenesis arises from excessive excitability in hyper-excitable neurons and glial cells, which synchronize and spread to previously normal, non-epileptogenic tissue. While there is clinical use of various anti-epileptic drugs to manage acute seizures, no effective treatment currently exists to halt the progression of epilepsy.

Experimental studies provide strong evidence for SF’s neuroprotective and anticonvulsant properties. In animal models, the combination of SF and the antioxidant N-acetylcysteine has been evaluated. SF was shown to activate Nrf2 in the brain following injury, triggering neuroprotective responses. These responses included reducing neuronal death, enhancing antioxidant defenses, and potentially modifying the progression of disease in a rat model of temporal lobe epilepsy [267].

Neurological insults, such as status epilepticus (SE), a condition involving prolonged, continuous seizures that are relatively uncommon among epilepsy patients [268], can be induced in experimental rodent models. In these models, SE triggers oxidative stress and excessive production of reactive oxygen species (ROS), both of which play a crucial role in the development of acquired epilepsy. SF’s direct benefits on mitochondrial function suggest potential anti-seizure activity in humans. Carrasco-Pozo et al. examined SF’s effects on hippocampal mitochondrial bioenergetics in mouse seizure models and found that SF enhanced ATP-linked respiration and state III respiration in mitochondria from both SE and non-SE mice [269]. This increase in energy production may reinforce SF’s antioxidant and anticonvulsant properties by supplying more ATP for essential cellular functions and defense mechanisms.

Recent findings further support SF’s impact on oxidative stress and mitochondrial dysfunction in epilepsy. Folbergrova et al. reported that SF mitigates oxidative stress and counteracts persistent deficiencies in complex I activity observed during and after SE in a rat model [270]. Similarly, Daněk et al. highlighted SF’s ability to ameliorate metabolic disturbances associated with epileptogenesis in multiple animal models of acquired epilepsy [271].

Together, these studies position SF as a promising candidate for epilepsy therapy. By targeting oxidative stress, mitochondrial dysfunction, and neuronal survival, SF may offer a novel approach to modifying disease progression and improving patient outcomes.

### 3.5. Sleep Disorders

Sleep disorders which include sleep apnea, circadian disruption, sleep disordered breathing and insomnia are closely linked to a variety of health conditions including neurodegenerative disease, cognitive impairment, and dementia [272].

A randomized, double-blind, parallel-group, placebo-controlled study was conducted to explore the effects of SF on sleep quality in Japan, where 1 in 5 adults are reported to have insomnia, and 50% of adults are unsatisfied with their sleep [273]. Healthy adults (*n* = 18) took 30 mg glucoraphanin or placebo for 4 weeks and there were significant increases in salivary melatonin and improvements in markers of inflammation. The increases in melatonin were hypothesized to be linked to the enhanced sleep quantity and quality as reported in visual analog scale questionnaires.

In a rodent model of obstructive sleep apnea-hypopnea, effects of SF intervention were examined [274]. SF alleviated hippocampal neuron apoptosis and neurocognitive dysfunction associated with chronic hypoxia. Their hypothesis was that by reducing the chronic intermittent hypoxia associated with sleep apnea, improvements in cognitive function such as working memory errors, reference memory errors, and total memory errors might be significant in the mouse test.

Use of a semisynthetic Nrf2 inducer (dimethyl fumarate) has demonstrated efficacy and reduced systemic inflammation in an obstructive sleep apnea clinical study [275], which suggests that there may be a similar role for SF. Further support has now been provided in a mouse model in which SF was protective against the neuroinflammation (related to endoplasmic reticulum stress) and cognitive impairment brought on by chronic intermittent hypoxia and sleep fragmentation [276]. These findings suggest a potential role for SF in mitigation of the sequelae of sleep apnea in human beings.

Aberrant circadian clock regulation has been associated with rhythmic activation of the Nrf2-GSH-mediated antioxidant defense pathway and a timed administration of SF significantly blocked this phenotype in a mouse, bleomycin-induced model [277].

## 4. Concluding Remarks

Sulforaphane is a natural product—a phytochemical component of cruciferous vegetables that has been studied for over three decades for its chemoprotective properties. Its biomedical effects have been examined in thousands of studies, including over 125 clinical interventions [278]. A significant portion of these focus on brain and CNS disorders, as discussed in Section 3 of this review and are listed in Supplementary Table S1. In addition to highlighting those disorders, Section 2 delves into the specific pathways and mechanisms through which SF impacts brain health. A closer look at these pathways and mechanisms reveals that many also underlie the beneficial systemic effects of SF throughout the body. This review highlights an urgent takeaway driven by two converging factors: (1) evidence-based dietary (and behavioral) habits are so critical to healthspan, and (2) brain development occurs early in life (Figure 1) and is crucial for long-term health. In light of this intersection, we emphasize the critical importance of early attention to optimal phytochemical intake (e.g., SF), alongside essential macronutrients, minerals, and vitamins. Furthermore, it may be both prudent and beneficial to consider supplementing or increasing SF intake in connection with certain disorders. Since phytochemicals are relatively inexpensive compared to pharmaceuticals, one may increase one’s phytochemical intake with something simple as a change in grocery shopping habits. This underscores the urgency of better understanding their appropriate use and dosing patterns as we strive to extend effective health, wellness, and preventive care in a responsible manner.

## Figures and Tables

**Table 1:** Representative proteins for which the genes are transcriptionally regulated by Nrf2, and a range of their half-lives (table adapted from [37]).

Representative Proteins	Half-Life
Biotransformation and detoxification including: AHR, CYP1B1, UGT1A1, GSTMQ, ABCB6, ABCC1, CBR1, EPHX1, ALDH3A2, ADH7, NQO1	8–87 h
Antioxidant enzymes, including: GPX4, GSR1, TXN1, PRDX1, SRXN1	9–54 h
Carbohydrate metabolism, including: G6PD, PGD, TALD01, TKT, ME1, UGDH	37–363 h
Lipid Metabolism, including: ACO7, ACOX1, SCD2, NFE2L2	0.3–149 h
Haem and iron metabolism, including: HMOX1 (HO-1), BLVRA, BLVRB, FECH, FTH1, FTL1	0.3–149 h
Mediators of inflammation, including: PLA2G7, PTGR1, CEBPB	6–91 h
Protein recycling and turnover, including: PLAMP2, SQSTM1, PSMB1	6–133 h
Regulation of GSH synthesis, including: GR, GPO, GST, GCL	18–45 h

**Table 3:** Disorders and other conditions in which there is sufficient evidence for an effect of SF, to discuss herein. We have focused upon conditions for which there is clinical evidence, and we have chosen not to discuss those at the end of the list for which there may be suggestive evidence, but not scientific consensus of any sort.

Disorder
*Discussed in this review*
alcohol-related disorders (including frank toxicity and FAS)
Alzheimer’s disease (AD)
autism spectrum disorder (ASD)
cognitive function and memory impairment
diabetes-related cognitive decline
epilepsy
Huntington’s disease
ischemia/reperfusion-induced brain injury
mood disorders, depression, and anxiety
Parkinson’s disease (PD)
pathologies from environmental and food-borne toxins
schizophrenia
sleep/circadian disorder(s)
stroke
traumatic brain injury (TBI) and spinal cord injury (SCI)
*Not further discussed herein*
amyotrophic lateral sclerosis
cerebral palsy
Friedreich’s ataxia
Herpes-encephalitis-induced neurotoxicity
hyperammonemia-induced hepatic encephalopathy
multiple sclerosis
vascular dementia

## Supplementary Materials

The following supporting information can be downloaded at: https://www.mdpi.com/article/10.3390/nu17081353/s1, Table S1: Clinical trials reported on clinicaltrials.gov with the keywords “sulforaphane” or “broccoli” or “glucoraphanin”, and filtered to include only those specifying neurologic and brain function.

## Abbreviations

The following abbreviations are used in this manuscript:

ABC	Aberrant Behavior Checklist
Ach	acetylcholine
AchE	acetylcholineesterase
AD	Alzheimer’s Disease
ADH	alcohol dehydrogenase
AGE	advanced glycation endproducts
ALDH	aldehyde dehydrogenase
AQP4	aquaporin 4
ASD	autism spectrum disorder
Aβ	amyloid-beta
BBB	blood–brain barrier
Bcl	anti-apoptotic B-cell lymphoma-2
HSP	heat shock protein
BDNF	brain-derived neutrophic factor
CHIP	carboxy-terminal Hsp70-interacting protein
CNS	central nervous system
COX-2	cyclooxygenase-2
DNMT	DNA methyl transferase
FAS	fetal alcohol syndrome
GCL	γ-glutamylcysteine ligase
GCLC	γ-glutamylcysteine ligase catalytic subunit
GCLM	γ-glutamylcysteine ligase modifier subunit
GGT	γ-glutamyl transferase
GPx	glutathione peroxidase(s)
GR	glucoraphanin
GS	glucosinolates
GSH	glutathione
GSSG	glutathione disulfide
GST	glutathione *S*-transferase(s)
HDAC	histone deacetylase
HMOX	heme oxygenase
HO-1	heme oxygenase-1
HSR	heat shock response
ICH	intracerebral hemorrhage(s)
IL-1β	interleukin-1 beta
IL-6	interleukin-6
IP-10	interferon-gamma inducible protein 10
ITC	isothiocyanate(s)
Keap1	Kelch-like ECH-associated protein 1
KO	knock-out
MATRICS	Measurement and Treatment Research to Improve Cognition in Schizophrenia
MCCB	MATRICS Consensus Cognitive Battery
mHtt	mutant huntingtin
MIF	macrophage migration inhibitory factor
miRNA	microRNA
MPO	myeloperoxidase(s)
MPTP	methyl-4-phenyl-1,2,3,6-tetrahydropyridine
NF-kB	nuclear factor kappa B
NMDA	*N*-methyl-D-aspartate
NQO1	NAD(P)H quinone dehydrogenase 1 or NAD(P)H dehydrogenase, quinone 1
Nrf2	NF-E2 p45-related factor 2
OARS	Ohio State University (OSU) Autism Rating Scale
PANSS	Positive and Negative Syndrome Scale
PCP	phencyclidine or phenylcyclohexyl piperidine
PD	Parkinson’s Disease
PGE2	protaglandin E2
Prx	peroxiredoxin
ROS	reactive oxygen species
SCFA	short-chain fatty acids
SCI	spinal cord injury
SF	sulforaphane
SOD	superoxide dismutase
SRS	Social Responsiveness Scales
TBI	traumatic brain injury
TCA	tricarboxylic acid cycle (also known as. Krebs cycle)
TNF-α	tumor necrosis factor alpha
Trx	thioredoxin
