# Supplementary material for: Sulforaphane and Brain Health: From Pathways of Action to Effects on Specific Disorders
Source: Nutrients. 2025 Apr 15;17(8):1353. doi: 10.3390/nu17081353 (PMC12030691; doi:10.3390/nu17081353)
Supplement: Supplementary file 1 [file nutrients-17-01353-s001.zip › nutrients-3578615-supplementary.pdf]

Table S1. Clinical trials reported on clinicaltrials.gov with the keywords “sulforaphane” or “broccoli” or “glucoraphanin”, and filtered to include only those specifying neurologic and brain function. There were 173 studies overall, and this list contains 25 studies which are listed by ascending NCT number. With the exception of the last entry, all content listed herein is “as reported” by clinicaltrials.gov, accessed April 6, 2025, and has not been edited or verified by the authors of this paper.

NCT Number	Conditions	Study Title	Study Status	Last Update Posted
NCT01474993	Autism	Sulforaphane-rich Broccoli Sprout Extract for Autism	COMPLETED	9/12/2018
NCT01716858	Schizophrenia	An Open Study of Sulforaphane-rich Broccoli Sprout Extract in Patients With Schizophrenia	COMPLETED	7/30/2015
NCT02561481	Autism Spectrum Disorder	Sulforaphane Treatment of Children With Autism Spectrum Disorder (ASD)	COMPLETED	12/17/2020
NCT02618174	Eating Behaviour	Enhanced Broccoli Consumption After a Liking Norm and Vegetable Variety Message: Effects After a 24 Hour Delay.	COMPLETED	12/1/2015
NCT02654743	Autism	Open Label of Clinical Trial of Sulforaphane in Children With Autism	COMPLETED	3/6/2019
NCT02677051	Autism|Autistic Disorder|Autism Spectrum Disorder|Autistic Behavior	Sulforaphane in a New Jersey (NJ) Population of Individuals With Autism	ACTIVE_NOT_RECRUITING	11/18/2024
NCT02810964	Schizophrenia|Schizoaffective Disorder	Sulforaphane to Reduce Symptoms of Schizophrenia	COMPLETED	7/27/2021
NCT02879110	Autism Spectrum Disorder	A 12-weeks Study to Evaluate Sulforaphane in Treatment of Autism Spectrum Disorder	COMPLETED	7/30/2019
NCT02880462	Schizophrenia	A 6-month Study to Evaluate Sulforaphane add-on Effects in Treatment of Schizophrenia	COMPLETED	3/24/2020

NCT02909959	Autism Spectrum Disorder|Autistic Disorder|Neurodevelopmental Disorder|Childhood Developmental Disorders, Pervasive	Sulforaphane for the Treatment of Young Men With Autism Spectrum Disorder	COMPLETED	6/4/2020
NCT03451734	Schizophrenia|Metabolic Syndrome	Optimizing and Individualizing the Pharmacological Treatment of First-episode Schizophrenic Patients	COMPLETED	8/12/2021
NCT03932136	Clinical High Risk Syndrome of Psychosis	Decreasing Risk of Psychosis by Sulforaphane (DROPS Trial)	RECRUITING	10/31/2023
NCT04213391	Alzheimer Disease	Effects of Sulforaphane in Patients With Prodromal to Mild Alzheimer's Disease	UNKNOWN	5/12/2020
NCT04246905	Depressive Disorder	A 12-weeks Study to Evaluate Sulforaphane in the Treatment of Depression	UNKNOWN	8/20/2020
NCT04252261	Frontal Lobe Dysfunction	Effects of Sulforaphane on Cognitive Function in Patients With Frontal Brain Damage	UNKNOWN	5/19/2020
NCT04521868	Schizophrenia	A 6-month Study to Evaluate Sulforaphane Effects in Schizophrenia Patients	COMPLETED	4/26/2023
NCT04805957	Autism|Autism Spectrum Disorder|Autistic Disorder	Validation Digital Bio-markers During Sulforaphane Treatment.	ENROLLING_BY_INVITATION	6/20/2024
NCT05084365	Parkinson Disease	A 6-month Study to Evaluate Sulforaphane Effects in PD Patients	RECRUITING	7/6/2023
NCT05145270	Major Depressive Disorder	A Comparative Study on Efficacy and Safety of add-on Sulforaphane or rTMS to Escitalopram for Major Depressive Disorder With Poor Response to Initial Treatment	UNKNOWN	12/6/2021
NCT05148169	Major Depressive Disorder	A Pilot Study on Effect of add-on Sulforaphane to SSRIs and Application of Niacin Skin Flush Response Test in Major Depressive Disorder	UNKNOWN	12/8/2021

NCT05233579	Fragile X Associated Tremor/Ataxia Syndrome (Fxtas) (Diagnosis)	Open-Label Trial of Sulforaphane in Premutation Carriers With FXTAS	COMPLETED	7/12/2023
NCT05848336	Autism Spectrum Disorder|Ophthalmic Abnormalities	The Gluten-Casein-free Diet in Children With Autism: A Clinical Results of the Ophthalmic and Behavioral Manifestations	COMPLETED	5/8/2023
NCT06491836	Attention Deficit Disorder	Oligoantigenic Diet and Core Symptomatology of Children With Attention-Deficit /Hyperactivity Disorder (ADHD).	NOT_YET_RECRUITING	8/12/2024
NCT06594536	Attention Deficit Disorder	Study of the Efficacy of Sulforaphane in Children Aged 6 to 12 With Attention Deficit Disorder With or Without Hyperactivity	NOT_YET_RECRUITING	9/19/2024
not listed	Schizophrenia	An Open Study of Sulforaphane-rich Broccoli Sprout Extract in Patients With Schizophrenia	completed	2015 publication
